# A High Precision Machine Learning-Enabled System for Predicting Idiopathic Ventricular Arrhythmia Origins

**DOI:** 10.3389/fcvm.2022.809027

**Published:** 2022-03-11

**Authors:** Jianwei Zheng, Guohua Fu, Daniele Struppa, Islam Abudayyeh, Tahmeed Contractor, Kyle Anderson, Huimin Chu, Cyril Rakovski

**Affiliations:** ^1^Schmid College of Science and Technology, Chapman University, Orange, CA, United States; ^2^Arrhythmia Center, Ningbo First Hospital, Zhejiang University, Ningbo, China; ^3^Interventional Cardiology, Loma Linda University Health, Loma Linda, CA, United States

**Keywords:** ventricular tachycardia, premature ventricular complex, catheter ablation, machine learning, ECG

## Abstract

**Background:**

Radiofrequency catheter ablation (CA) is an efficient antiarrhythmic treatment with a class I indication for idiopathic ventricular arrhythmia (IVA), only when drugs are ineffective or have unacceptable side effects. The accurate prediction of the origins of IVA can significantly increase the operation success rate, reduce operation duration and decrease the risk of complications. The present work proposes an artificial intelligence-enabled ECG analysis algorithm to estimate possible origins of idiopathic ventricular arrhythmia at a clinical-grade level accuracy.

**Method:**

A total of 18,612 ECG recordings extracted from 545 patients who underwent successful CA to treat IVA were proportionally sampled into training, validation and testing cohorts. We designed four classification schemes responding to different hierarchical levels of the possible IVA origins. For every classification scheme, we compared 98 distinct machine learning models with optimized hyperparameter values obtained through extensive grid search and reported an optimal algorithm with the highest accuracy scores attained on the testing cohorts.

**Results:**

For classification scheme 4, our pioneering study designs and implements a machine learning-based ECG algorithm to predict 21 possible sites of IVA origin with an accuracy of 98.24% on a testing cohort. The accuracy and F1-score for the left three schemes surpassed 99%.

**Conclusion:**

In this work, we developed an algorithm that precisely predicts the correct origins of IVA (out of 21 possible sites) and outperforms the accuracy of all prior studies and human experts.

## Introduction

Premature ventricular complexes (PVC) are commonly found in persons without evidence of structural heart disease. However, in a subset of patients, they may herald the presence of underlying cardiomyopathy. A daily high burden of PVC or paroxysmal ventricular tachycardia (VT) may cause a cardiac chamber dilation and an impairment of the systolic function of the heart a condition known as PVC-induced cardiomyopathy. In a population-based study (1) of older adults without any heart failure signs or systolic dysfunction, the baseline of PVC/VT burden was significantly associated with an adjusted increased odds of decreased left ventricular ejection fraction (odds ratio (OR), 1.13; 95% confidence interval (CI), 1.05–1.21) and an increased adjusted risk of incident heart failure (hazard ratio (HR), 1.06; 95% CI, 1.02–1.09) and death (HR, 1.04; 95% CI, 1.02–1.06). Idiopathic ventricular arrhythmias (IVA) is a term commonly used to describe PVC or VT in the absence of structural heart disease. Catheter ablation (CA) is an efficient treatment with a class I indication for IVA when drugs are ineffective or have unacceptable side effects (2–4). Complications of catheter ablation include cardiac perforation, tamponade, and coronary artery occlusion (5). The success of the CA procedure is predicated on a precise and fast determination of the correct ablation sites. The origins of PVC and VT can be identified through analysis of ECGs, activation mapping, pace mapping, and echocardiogram. Using standard 12-lead ECGs, an accurate prediction of the origins of IVA before the ablation procedure can optimize the CA treatment strategy, reduce ablation duration, and avoid operative complications (6). Previous studies have proposed several ECG criteria and models for the estimation of IVA origins at different levels of the anatomical structure. For instance, Zheng et al. (7), Di et al. (8), Cheng et al. (9), He et al. (10), Xie et al. (11), Efimova et al. (12), Yoshida et al. (13), Nakano et al. (14), Cheng et al. (15), Betensky et al. (16), Arya et al. (17), and Kamakura et al. (18) proposed an algorithm or index to predict origins from right or left outflow tract; (19) developed an ECG criteria for locations under outflow tract; (20, 21) devised an ECG criteria for locations under right ventricular outflow tract; (22) proposed transitional zone index to separate origins from right ventricular outflow tract and aortic sinus cusp. However, these results have been limited by analyzing only a few locations, ECG measurement efficiency, and the ability to deploy the model to real-life medical settings. For instance, the majority of prior works were only focused on locating general regions, such as left outflow tract, right outflow tract, non-outflow tract, etc., rather than taking into account all possible ablation sites available in a systemic perspective. Moreover, the decision criterion derived from ECG measurement, such as transitional index and ratios of QRS complex altitudes, are not available in the commercial software and open-source tools. Thus, operators are reluctant to apply prior study results since measurement and computation of these criteria are cumbersome manual works. In contrast, we developed an optimal multi-stage approach that automatically extracts features from standard 12-lead ECGs and incorporates these features into a machine learning classification model to predict the precise site of IVA origin with fewer limitations and higher accuracy than prior studies. In the common clinical practice, the estimation of origins will be performed with a hierarchical sequence from general regions, such as right and left ventricular, to detailed locations. Therefore, the study encompassed four classification schemes to classify IVA origin sites from 3 general regions to 21 anatomical sites. We also provided a multi-prospective analysis for revealing the underlying relationship between important ECG characteristics and the sites of IVA origins.

## Methods

### Study Design

The institutional review board of Ningbo First Hospital of Zhejiang University has approved this retrospective study and granted the waiver of consent requirement. The study was conducted in accordance with the Declaration of Helsinki. The estimation of origins with a hierarchical sequence from general regions to detailed locations can cater to different level demands of the application. Therefore, we designed four classification schemes with increasing numbers of anatomical origin sites. The first scheme will help the operators to figure out the origin from epicardium of left ventricular summit, right, and left ventricle. The second one can separate origins from left/right outflow tract and left/right non-out flow tract, respectively. The third one is able to predict 18 anatomical locations, and the fourth scheme can distinguish 21 possible sites. The origin sites of each scheme are shown in Table 1.

**Table 1:** Performance report with 95% CIs and hierarchical anatomy structure for four classification schemes.

	**Scheme 1**	**Scheme 2**	**Scheme 3**	**Scheme 4**
Accuracy (95% CI)	99.79 (99.41–99.89)	99.62 (99.09–99.78)	97.78 (96.76–98.41)	98.24 (97.36–98.71)
F1-score (95% CI)	99.84 (99.6–99.96)	99.42 (98.79–99.75)	97.74 (94.15–99.73)	98.56 (97.88–99.12)
Anatomical sites	LV endocardium	Outflow tract	LCC	LCC
			RCC	RCC
			AMC	AMC
			Summit	Summit
			LCC-RCC Commissure	LCC-RCC Commissure
		Non-Outflow tract	Left His bundle	Left His bundle
			MV	MV
			Left Septal	LAF
				LPF
			Papillary Muscle	LAPM
				LPPM
	RV endocardium	Outflow tract	AC	AC
			LC	LC
			RC	RC
			RVOT septal	RVOT posterior septal
				RVOT anterior septal
			RVOT free wall	RVOT free wall
		Non-Outflow tract	Right His bundle	Right His bundle
			TV	TV
			RAPM	RAPM
	Epicardium of LV summit	Epicardium of LV summit	Epicardium of LV summit	Epicardium of LV summit

*LC, left cusp; LCC, left coronary cusp; AC, anterior cusp; RC, right cusp; AMC, aortomitral continuity; LPF, left posterior fascicle; TV, tricuspid valve; LAF, left anterior fascicle; LPPM, left posterior papillary muscle; MV, mitral valve; LAPM, left anterior papillary muscle; RCC, right coronary cusp; RAPM, right anterior papillary muscle*.

For each patient, we extracted all available ECG recordings when PVC or VT occurred. According to the window size setting (introduced in the section of ECG measurement protocol), each recording included one QRS complex and possibly contained segments before the Q-wave onset and after the S-wave offset. According to the outcome of ablation, each ECG recording was labeled by the anatomical origins that represented successful ablation sites. To fairly evaluate the estimation performance of proposed work, this study employed a training-validation-testing design that randomly and proportionally assigned all participated patients into distinct cohorts. Therefore, training, validation, and testing cohorts did not have ECG recording samples from the same patient. However, since each patient could have a different length of ECG recordings when PVC or VT occurred, the training cohort did not have equal numbers of ECG recordings associated with individual anatomical sites. Thus, we employed an oversampling method to increase minority samples in the training cohort to address the imbalanced learning problem. For instance, the number of patients with epicardium of left ventricular (LV) summit origins was five (shown in column 2 of Table 2, rather than 10 that is the number of ECG recordings (shown in column 3 of Table 2). In the first step, we assigned three patients to the training cohort, one patient to the validation cohort, and one patient to the testing cohort. Secondly, cardiologists in the research group selected three ECG recordings for the patient in the testing cohort. Proportionally, we extracted three ECG recordings for the patient in the validation cohort and 25 ECG recordings for the patients in the training cohort. Finally, we achieved 80–10–10% distribution split across training, validation, and testing cohorts both per patient and per ECG recordings. During the training stage, the oversampling method was implemented to increase the number of ECG recordings from 25 to 1,694, the largest sample number associated with left cusp (LC) origin. The overall sample distributions of patients and ECG recordings for classification scheme 4 are presented in Table 2.

**Table 2:** Sampls distribution of classification scheme 4.

**Locations**	**Patients, n (%)**	**ECG recordings, n (%)**	**Training cohort, n (%)**	**Validation cohort, n (%)**	**Testing cohort, n (%)**
LC	67 (15.12)	2,118 (14.36)	1,694 (80)	212 (10)	212 (10)
RVOT posterior septal	43 (9.93)	1,848 (12.53)	1,478 (80)	185 (10)	185 (10)
LCC	41 (9.47)	588 (3.99)	470 (80)	59 (10)	59 (10)
AC	38 (8.78)	1,079 (7.31)	863 (80)	108 (10)	108 (10)
RVOT free wall	32 (7.39)	1,287 (8.72)	1,029 (80)	129 (10)	129 (10)
RVOT anterior septal	32 (7.39)	1,014 (6.87)	812 (80)	101 (10)	101 (10)
Right His bundle	31 (7.16)	705 (4.78)	563 (80)	71 (10)	71 (10)
RC	24 (5.54)	353 (2.39)	283 (80)	35 (10)	35 (10)
AMC	23 (5.31)	1,434 (9.72)	1,146 (80)	144 (10)	144 (10)
LPF	18 (4.16)	913 (6.19)	731 (80)	91 (10)	91 (10)
TV	18 (4.16)	563 (3.82)	451 (80)	56 (10)	56 (10)
LAF	13 (3)	885 (6)	707 (80)	89 (10)	89 (10)
LCC-RCC commissure	11 (2.54)	172 (1.17)	138 (80)	17 (10)	17 (10)
Epicardium of LV summit	10 (2.31)	351 (2.38)	281 (80)	35 (10)	35 (10)
LPPM	9 (2.08)	677 (4.59)	541 (80)	68 (10)	68 (10)
MV	8 (1.85)	330 (2.24)	264 (80)	33 (10)	33 (10)
LAPM	8 (1.85)	192 (1.3)	154 (80)	19 (10)	19 (10)
RCC	7 (1.62)	51 (0.35)	41 (80)	5 (10)	5 (10)
Summit	5 (1.15)	31 (0.21)	25 (80)	3 (10)	3 (10)
Left His bundle	3 (0.69)	149 (1.01)	119 (80)	15 (10)	15 (10)
RAPM	2 (0.46)	14 (0.09)	12 (80)	1 (10)	1 (10)

*LC, left cusp; LCC, left coronary cusp; AC, anterior cusp; RC, right cusp; AMC, aortomitral continuity; LPF, left posterior fascicle; TV, tricuspid valve; LAF, left anterior fascicle; LPPM, left posterior papillary muscle; MV, mitral valve; LAPM, left anterior papillary muscle; RCC, right coronary cusp; RAPM, right anterior papillary muscle*.

**Figure 1:**
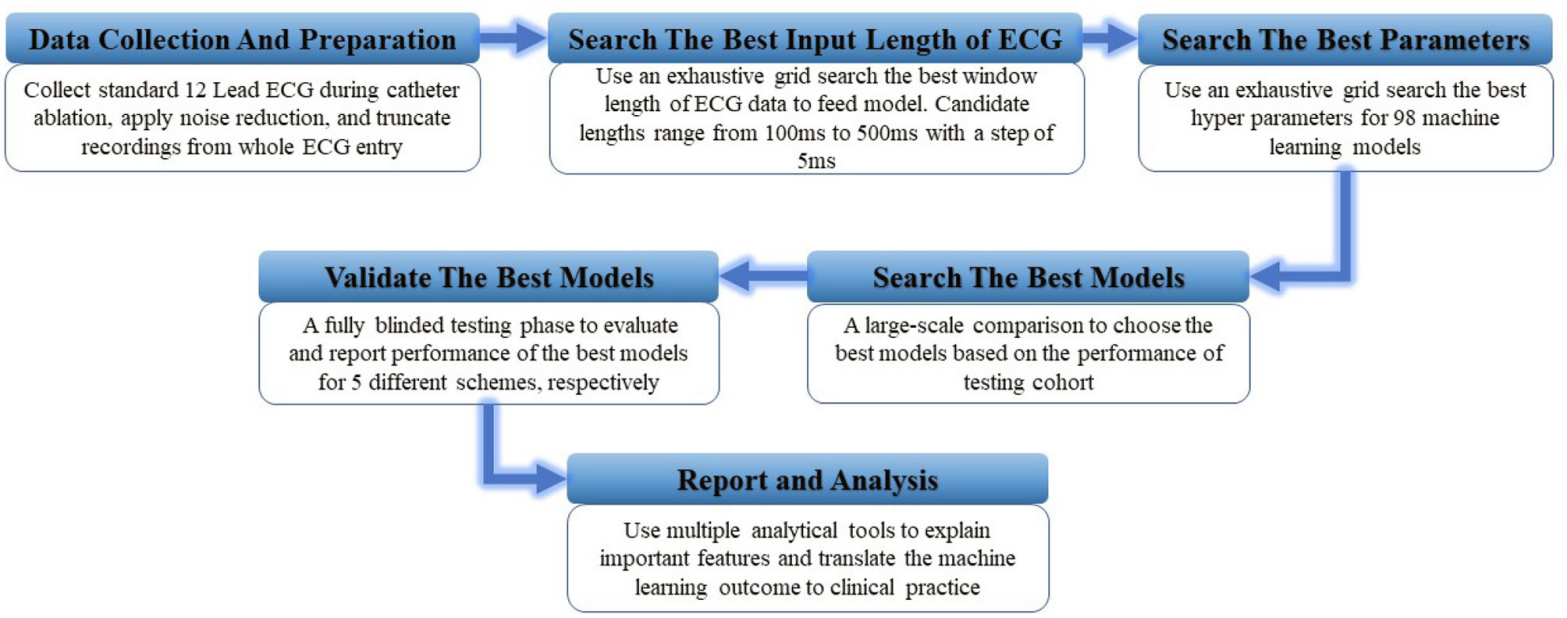
The illustration of study design. A total of six experiment steps were conducted sequentially.

For every classification scheme presented in Table 1, a four-stage experiment was conducted sequentially: (1) a random forest model to perform an exhaustive search for the best window size of ECG input data; (2) a hyper-parameters grid search for 98 competing machine learning models based on validation data performance [a design that has been successfully implemented in a similar study (23)]; (3) a comprehensive comparison of all 98 models to identify the best model based on the accuracy achieved in the testing cohort; and (4) a fully blind testing phase to evaluate, interpret, and report the best model performance. The pipeline of this experiment is presented in Figure 1.

### Patient Selection

We analyzed ablation procedure logs and image recording files, including MRI, ECG, Cardio Echo, *X*-ray, and 3D mapping, of a total of 747 patients who had undergone mapping and ablation for IVA at the Ningbo First Hospital of Zhejiang University from March 2007 to September 2019. A 12-lead surface ECG test was obtained on each clinic visit, and a 24-h Holter monitoring was recorded before the ablation. A requirement for entry into the study was for patients to experience the burden of IVA for greater than 10% of the total duration of time.

**Figure 2:**
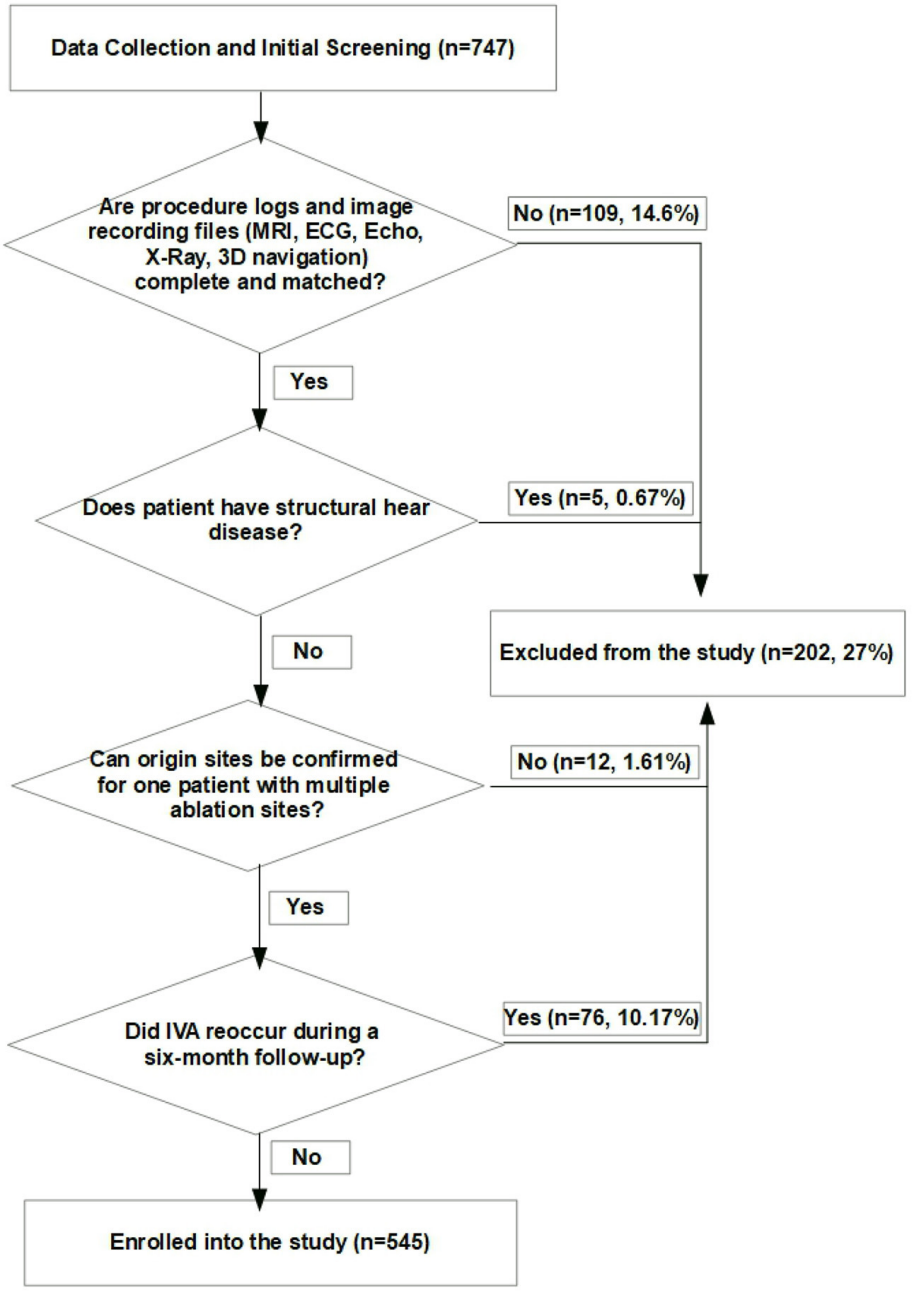
Decision process to exclude patients from the study.

A total of 109 (14.6%) patients were removed from the study since ablation logs and image recordings were not complete or mismatched. If IVA burden was less than 5% of the total test duration of the Holter monitoring and there was no recurrence of clinical IVA during clinic visit in the first six-month follow-up after CA, the ablation was regarded as a successful procedure, and origin sites of IVA were ascertained. A total of 76 (10.17%) patients were excluded from this study because of the recurrence of IVA in the first six-months of follow-up. Moreover, the patients who had coronary artery disease, myocarditis, cardiomyopathy, and ischemic heart disease were removed from the study according to the clinical history, echocardiograms, cardiac magnetic resonance, angiography and coronary computerized tomography (CT). A summary description of the demographics and clinical features are shown in Table 3 and five patients were removed from the study because of conditions mentioned above. In addition, 12 patients (1.61%) who had multiple ablation sites were excluded from the study since origin sites could not be precisely confirmed according to the rules defined in the section of Origin sites confirmation. Finally, a total of 545 patients with anatomically normal hearts without congenital disease or prior operations were included in this study. A diagram (shown in Figure 2) precisely presents the patient selection process.

**Table 3:** Baseline clinical characteristics of the patients.

**Characteristic**	**Total**	**Training cohort**	**Validation cohort**	**Testing cohort**	**P-value**
Patients, *n* (%)	545	436 (80)	55 (10)	54 (10)	NA
Age, Mean ± SD, year	47.1 ± 12.6	46.8 ± 12.3	47.5 ± 13.4	45.7 ± 15.4	0.92
Male, *n* (%)	178 (32.66)	144 (33)	16 (29.1)	18 (32.73)	0.949
BMI, Mean ± SD, (kg/m^2^)	29.20 ± 4.18	29.05 ± 4.67	27.68 ± 3.12	28.08 ± 5.21	0.089
Cardiac MRI, *n* (%)	10 (1)	9 (1)	1 (0)	0 (0)	NA
Angiography, *n* (%)	6 (1)	6 (1)	0 (0)	0 (0)	NA
Coronary CT, *n* (%)	12 (2)	6 (1)	5 (1)	1 (0)	0.016
Clinical findings on entry					
History of hypertension, *n* (%)	54 (10)	33 (6)	13 (2)	8 (1.5)	<0.01
History of diabetes, *n* (%)	37 (6.7)	30 (5.5)	2 (0)	5 (1)	0.712
Antiarrhythmic drugs, *n* (%)	225 (41.3)	205 (37.6)	9 (1.7)	11 (2)	<0.01
Echocardiogram test					
Echocardiograms, *n* (%)	187 (34.3)	136 (25)	33 (6.1)	18 (3.3)	<0.01
LV internal systolic dimension, Mean ± SD, mm	33.6 ± 4.1	33.1 ± 3.3	30.9 ± 4.7	32 ± 2.9	0.288
LV internal diastolic dimension, Mean ± SD, mm	49.9 ± 6.1	50.3 ± 5.2	49.1 ± 6.6	48.5 ± 6.7	0.099
lnterventricular septal thickness, Mean ± SD, mm	11.2 ± 1.7	11.3 ± 1.7	11.2 ± 2	11.1 ± 1.4	0.772
LV posterior wall thickness, Mean ± SD, mm	10.7 ± 1.2	10.5 ± 9	11.1 ± 1.2	10.7 ± 0.7	0.842
RV outflow tract, Mean ± SD, mm	31.9 ± 4.7	30.7 ± 5.5	33.7 ± 4.4	30.6 ± 4.5	0.647
LVEF (%)	60.8 ± 5.5	61.4 ± 4.8	59.0 ± 4.4	61.9 ± 3.5	0.289
ECG					
PVC burden (n/24h Holter), Mean ± SD	27146.5 ± 10611.8	25667.2 ± 11320	27884.3 ± 11734.2	26724.4 ± 12128.5	0.592
Frequent PVC, *n* (%)	513 (94.13)	414 (94.95)	47 (85.45)	51 (94.44)	0.072
Paroxysm VT, *n* (%)	19 (3.49)	15 (3.44)	4 (7.27)	1 (1.85)	0.483
Persistent VT, *n* (%)	13 (2.39)	7 (1.61)	4 (7.27)	2 (3.7)	0.061
Prior CA, *n* (%)	5 (0.92)	2 (0.46)	1 (1.82)	2 (3.7)	0.065

*BMI, body mass index; MRI, magnetic resonance imaging; CT, computerized tomography; LV, left ventricular; LVEF, left ventricular ejection fraction. Frequent PVC means that PVC burden is above 10% of total test duration*.

### Classification of Anatomical Sites

**Figure 3:**
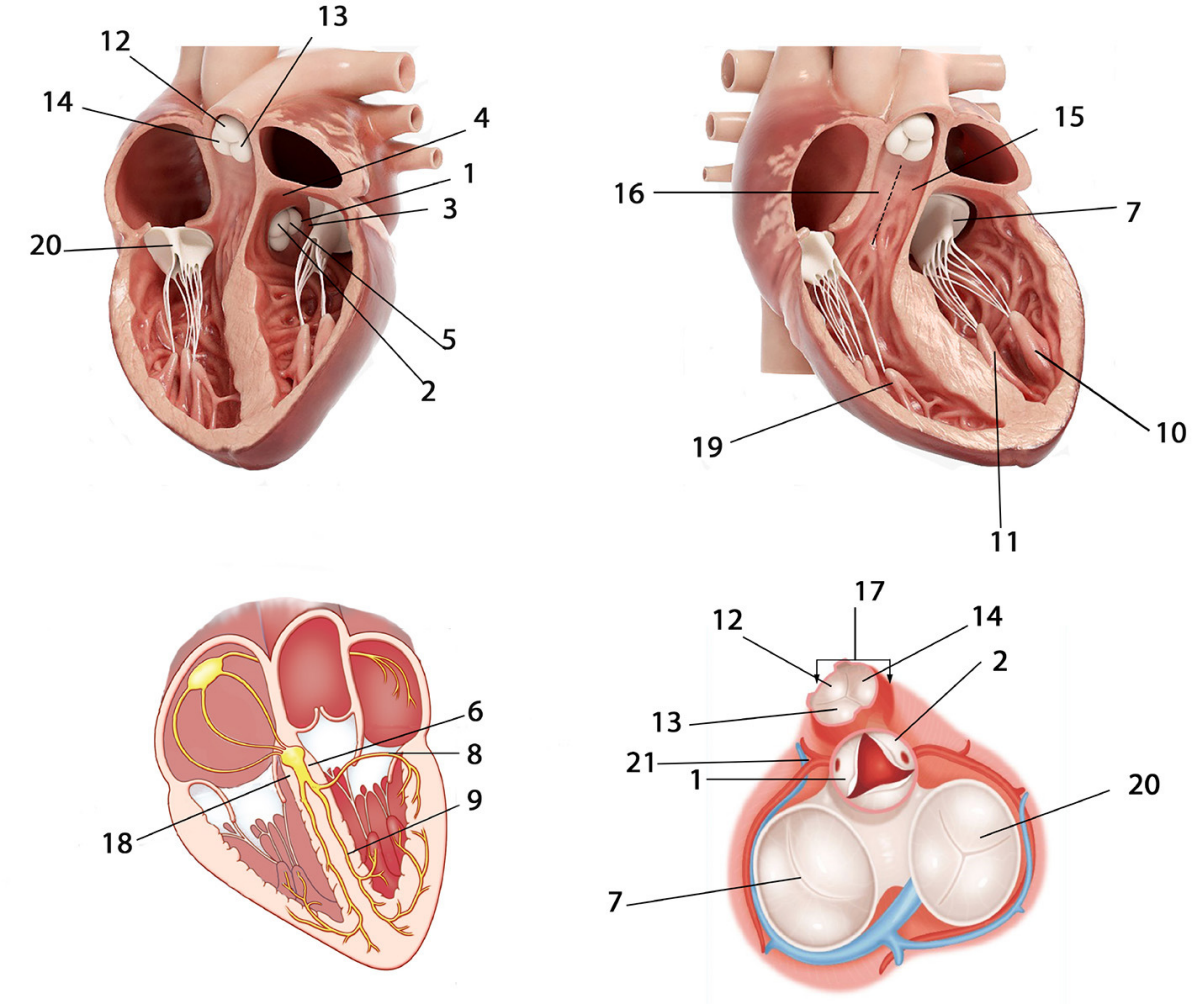
Anatomical locations of possible IVA origins. 1 = LCC; 2 = RCC; 3 = AMC; 4 = Summit; 5 = LCC-RCC commissure; 6 = Left His bundle; 7 = MV; 8 = Left anterior fascicle (LAF); 9 = Left posterior fascicle (LPF); 10 = Left anterior papillary muscle (LAPM); 11 = Left posterior papillary muscle (LPPM); 12 = AC; 13 = LC; 14 = RC; 15 = RVOT anterior septal; 16 = RVOT posterior septal; 17 = RVOT free wall; 18 = Right His bundle; 19 = Right anterior papillary muscle (RAPM); 20 = TV; 21 = Epicardium of LV summit. The abbreviations are as in Table 1.

The general regions of origin of IVA include left ventricular (LV) endocardium, right ventricular (RV) endocardium, and epicardium of LV summit. The origins in the RV and LV endocardium were anatomically divided into right ventricular outflow tract (RVOT), right ventricular non-outflow tract (RVNOT), left ventricular outflow tract (LVOT), and left ventricular non-outflow tract (LVNOT), respectively. The LVOT endocardium was divided into five regions: left coronary cusp (LCC), right coronary cusp (RCC), aortomitral continuity (AMC), summit, and LCC-RCC commissure. The LVNOT endocardium region was divided into left His bundle, mitral valve (MV), left septal including left anterior fascicle (LAF) and left posterior fascicle (LPF), and left papillary muscle including left anterior papillary muscle (LAPM) and left posterior papillary muscle (LPPM). The possible origin sites in the RVOT endocardium were identified by 3-dimensional directions: anterior and posterior, right and left, and superior and inferior. Accordingly, origins under the RVOT were classified into six regions: anterior cusp (AC), left cusp (LC), right cusp (RC), RVOT septal (including posterior septal and anterior septal), and free wall. The RVNOT endocardium region includes right His bundle, tricuspid valve (TV), and right anterior papillary muscle (RAPM). The site of MV/TV here could include anterior, posterior, and lateral MV/TV. Each location is depicted in Figure 3, and the classification of hierarchical anatomical sites is shown in Table 1.

### Catheter Ablation Procedures

**Figure 4:**
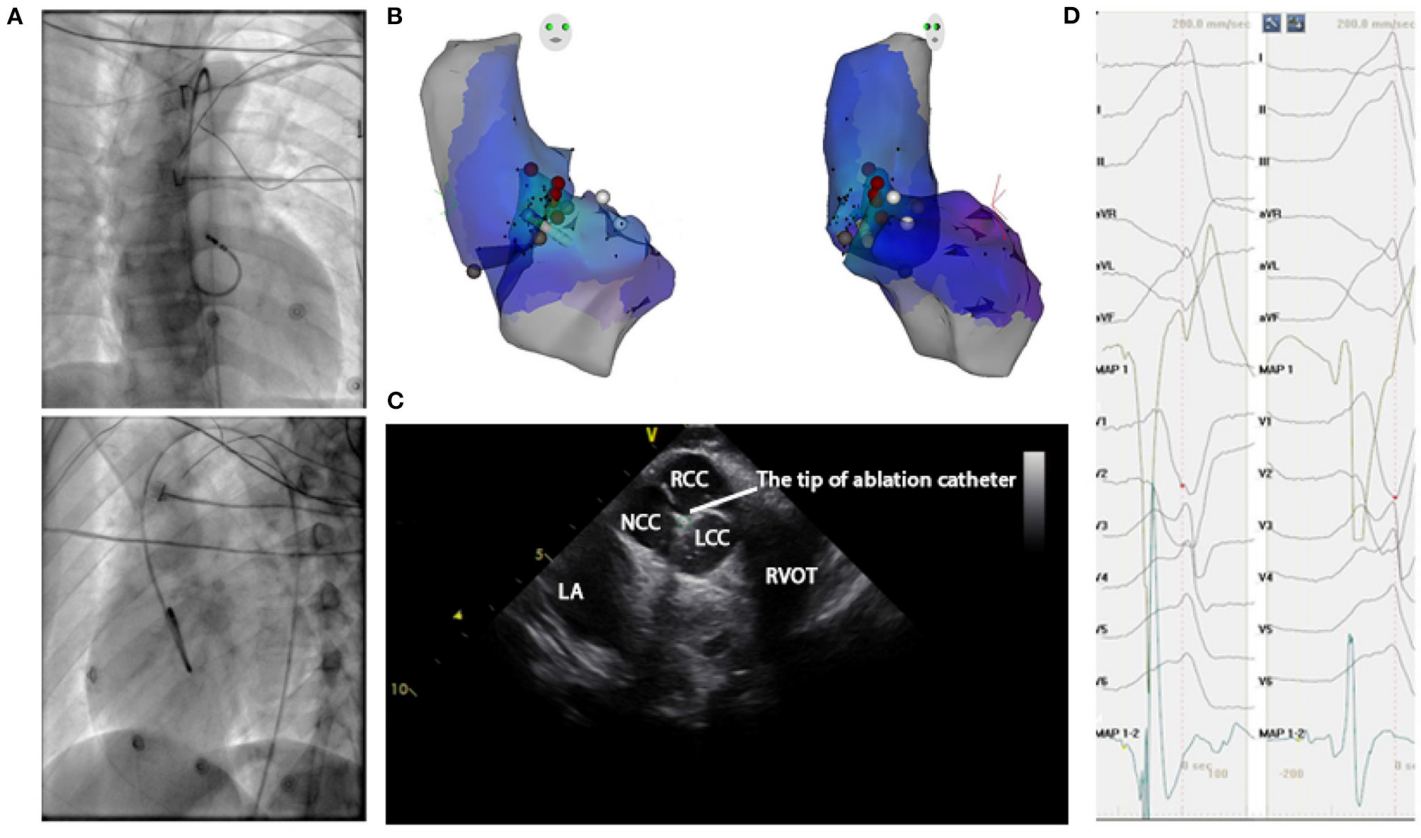
Mapping for IVA originating from LCC-RCC commissure in LVOT. **(A)** Right anterior oblique and left anterior oblique fluoroscopic views showed an ablation catheter in the LVOT. Ablation in the LVOT (LCC-RCC commissure) eliminated the PVC within 3 s. **(B)** The three-dimensional anatomic representation of the RV endocardium, LV endocardium, and venous system with the ablation catheter positioned at the anterior interventricular vein. **(C)** The green circle indicated the tip of ablation catheter in LCC-RCC commissure. **(D)** The earliest bipolar and unipolar activation time (–30 ms) are shown. MAP1 is unipolar signal and MAP1-2 is bipolar signal.

Administration of antiarrhythmic drugs had been stopped for at least five half-lives before the inception of the ablation procedure. A 4.0-mm 7F irrigated-tip ablation catheter (Navistar, Biosense Webster, Diamond Bar, CA) was initially placed in the RVOT for mapping. Both fluoroscopy and electroanatomic mapping systems (CARTO, Biosense Webster, Diamond Bar, CA, USA or NavX Velocity, St. Jude Medical, St. Paul, MN, USA) were used to localize the anatomical position of the catheter ablation for all cases in this study. For a total of 187 patients enrolled after 2018, the echocardiogram was used to identify specific anatomical structures such as cusps and papillary muscles. For instance, Figure 4 presented the fluoroscopy, 3-dimensional mapping, intracardiac echocardiography, and activation mapping for a patient who has VT originating from LCC-RCC commissure of LVOT. Using point by point mapping, anatomical aggregated maps were created. Activation mapping was performed in all patients during VT and PVC. Pace mapping was also performed, with the lowest pacing output (2 to 20 mA) and pulse width (0.5 to 10 ms), to capture the ventricular myocardium at the site of the earliest activation. If suitable ablation sites for the RVOT origin were not located or ablation failed to abolish the arrhythmia, extended mapping to the LVOT site was deployed via a retrograde aortic approach. After target sites were located, radiofrequency energy was delivered with maximum power up to 35 W with a saline irrigation flow rate of 17–9 ml/min and a maximum electrode-tissue interface temperature of 43°C. If the VT/PVC disappeared, or the frequency of arrhythmias diminished after the first 30 s of ablation, the energy was delivered continuously from 60 to 180 s. For PVC/VT that originated from summit under LVOT, we carried the ablation through LVOT endocardium by 35W power after successfully locating the optimal ablation target site. The acute ablation success was defined as the absence of spontaneous or induced IVA at 30 min after the last energy delivery and confirmed by continuous cardiac telemetry in the subsequent 24 h of inpatient care.

### Origin Sites Confirmation

In this study, the successful ablation sites that followed the above classification convention were outcome labels for the machine learning predictive models, and the corresponding 12-lead ECG data mainly containing a QRS complex were the inputs variables. One can map one-to-one relationship between the origin site (successful ablation site) and the QRS complex in most cases. However, for some sophisticated cases, if PVC or VT originated from one site, such as mid-myocardial layer, and had multiple PVC exit sites due to lesion biophysics, heat sink effect from adjacent vessels, fiber orientation and heat transfer, etc., multiple QRS morphologies would be presented. We enrolled such cases into the study when the following three criteria were satisfied: (1) the absence of spontaneous or induced PVCs or VT after energy delivered, (2) the confirmation of acute ablation success mentioned in the section of Catheter ablation procedures, and (3) the confirmation of successful sustained ablation mentioned in the section of Followup. For such cases, multiple QRS complex inputs will have single origin site label. In addition, if PVC or VT originated from multiple sites and the QRS complex will present different morphology, ablation needs to be carried out in multiple locations, maybe in different chambers. The operator will ablate the possible origin site according to QRS morphology of the most frequently occurred PVC or VT, one by one. The same criteria mentioned above were harnessed to confirm origin sites for such scenarios as well. Strictly speaking, we used the successful ablation site to estimate the PVC/VT origin site and utilized the ECG signals to predict the successful ablation site, rather than PVC exit site. Thus, origin sites in this study were the names of successful ablation sites shown in Table 1.

### Follow Up

All antiarrhythmic drugs were discontinued after ablation procedure and during the follow-up period. In the 24 h of inpatient care following the ablation procedure, every patient received continuous ECG monitoring. After discharge, the patients underwent a follow-up two weeks after the ablation. These follow-ups continued every month at the cardiology clinic thereafter. A 12-lead surface ECG tests were obtained at each clinic visit, and a 24-h Holter monitoring was prescribed at three and six months after the ablation. The successful sustained ablation was defined for patients that did not experience the recurrence of IVA during clinic visit and frequent PVC or VT (happening above 5% of total test duration) in the first six-months of follow-up.

### ECG Measurement Protocol

**Figure 5:**
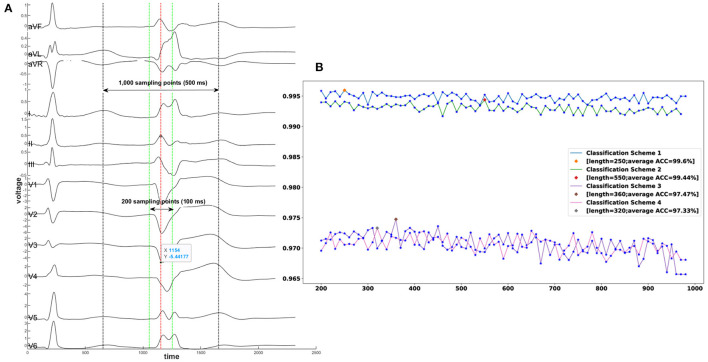
A demonstration of exhaustive grid search for the best window size of an ECG recording. **(A)** A segment of an ECG shows one sinus rhythm heartbeat and one PVC from a patient with PVC originating from the tricuspid valve. The exhaustive grid search using the ensemble random forest model gave the optimal length of an ECG recording to the highest prediction accuracy. The red dotted line aligned with the R-wave point at lead II represents the reference line. The range between the two green dotted lines is the minimum candidate with input length of 200 sampling points (100 ms). The range between the two black dotted lines is the maximum candidate of input length of 1,000 sampling points (500 ms). **(B)** *X*-axis denotes the window sizes of ECG data, and *Y*-axis denotes the average accuracy. The best window size for classification scheme 1 is 250 sampling points; for classification scheme 2–550 sampling points; for classification scheme 3–360 sampling points: for classification scheme 4–320 sampling points. ACC = Accuracy.

The 12-lead ECGs were collected at a sampling rate of 2,000 Hz using the standard electrode placement. In this work, the coif5 Wavelets and Stein's Unbiased Risk Estimator (SURE)-based threshold were implemented by a MATLAB (Natick, Massachusetts, The MathWorks Inc.) program for noise reduction. As machine learning model input, each ECG recording consisted of smoothed voltage values around one R-wave peak when PVC or VT happened (shown in Figure 5). To achieve the best classification performance and computational effectiveness, we implemented an exhaustive grid search using the ensemble random forest model to find the best window size of an ECG recording that achieved the highest accuracy of prediction. The candidate sizes ranged from 200 sampling data points (100 ms) to 1,000 sampling data points (500 ms) with a step of 10 sampling data points (5 ms). The reference line for each ECG recording is the R-wave peak point at lead-II (shown in Figure 5A). For the four classification schemes, Figure 5B showed the optimal window sizes of ECG recording that attained the best prediction performance based on the validation cohorts, window sizes consisting of 250 (125), 550 (275), 360 (180), and 320 (160) sampling points (ms), respectively. Moreover, the visualization validation was performed after optimal window sizes were obtained. For instance, the V1-lead ECGs that have labels of 21 sites predicted by scheme 4 were shown in Supplementary Figure 13.

### Optimal Strategies for Machine Learning Model Tuning

Our study aimed at designing the optimal machine learning algorithm consisting of multiple stages and data preprocessing. We carried out a comprehensive grid search to identify the optimal hyper-parameter values for each classification method presented in Supplementary Table 10. We selected, as optimal, the hyper-parameter values that attained the highest weighted average F_1_-Score on the validation cohort. Ensemble machine learning methods based on multiple samplings were used to improve classification performance. Two major stream ensemble methods, averaging and boosting, were implemented in this work. The first method consists of building numerous classifiers that are independently trained on different observed samples, and the individual results are averaged. This approach has the computational advantage of carrying out the independent training steps in parallel. In contrast, the gradient boosting method builds a set of classification models that will work sequentially. A boosting model was optimized and trained by feeding misclassified samples from a prior model until a quasi-optimal model with the lowest misclassification probability was obtained. In this work, we compared six ensemble methods, including Bagging Average, Random Forest, AdaBoost, Gradient Boost Tree, Extreme Gradient Boost Tree, and Extremely Randomized Tree.

We wrapped the ensemble methods and base classifiers into three meta-classifiers, One vs. Rest, One vs. One, and Error-Correcting Output-Codes. It is possible to use these meta-classifiers with ensemble algorithms hoping that their accuracy or runtime performance improves. A total of 98 different combinations were compared in this study, after combining meta-classifiers with ensemble methods and base classifiers that included Decision Trees, K Nearest Neighbors, Nearest Centroid, Gaussian Naive Bayesian, Multinomial Naive Bayesian, Complement Naive Bayesian, Bernoulli Naive Bayesian, Linear Classifier, Quadratic Discriminant Analysis, Multinomial Logistic Regression, Multilayer Perceptron Neural Net, Ridge Regression Classifier, Linear Classifiers with Stochastic Gradient Descent, Passive Aggressive Classifier, and Linear Support Vector Machine.

Convolutional neural network (CNN) models and recurrent neural network (RNN) models, such as long short term memory network (LSTM) with attention, have shown some promising results in ECG data analysis (24, 25). These models used sequential transformations of the raw data as features that were ultimately fed into a multinomial logistic regression classifier (softmax unit). Properly training them requires extensive data for each category outcome. A CNN with a 36 layer residual block structure and 32-layer long short term memory network with attention were also put into the comparison list.

After comparing all the above machine learning algorithms, our results showed that the Extremely Randomized Trees (26) classifier attained the highest performance in all settings, as evidenced by the achieved accuracy, F1-scores, and their 95% CIs.

### Statistical Analysis

For the continuous variable age, we calculated the mean and standard deviation. For the categorical variables, gender and locations, we calculated frequency counts and percentages. One-way Analysis of Variance (ANOVA) was used to test for differences of means across groups. Associations between categorical variables were analyzed with chi-square test and Fisher's exact test. A *p*-value < 0.05 was considered as statistically significant. The noise reduction and summary of the classification results *via* confusion matrices were done in MATLAB (Natick, Massachusetts, The MathWorks Inc.) Statistical optimization of the 98 models was done through iterative training using Python's scikit-learn package (27). The Seaborn package was employed to conduct univariate and multi-variate analysis for feature importance. The feature importance heat map was drawn by our proprietary Python (28) module. In the heat map, the X-axis denotes 12 leads, and the *Y*-axis indicates the sampling point. The ascending color brightness indicates the increasing variable importance magnitudes. The performances of all models were compared using the accuracy, sensitivity (SE), specificity (SP), positive predictive value (PPV), negative predictive value (NPV), balanced accuracy, adjusted accuracy, and the F1-Score measures. We used a two-sided 95% confidence interval (CI) to summarize the sample variability in the estimates. CIs were obtained by bootstrapping with 2,000 replications carried out in R version 3.5.3.

### Computation Resource

The computations and simulations were conducted in parallel on the Chapman University Schmid cluster with 500 cores and 1T RAM. The entire process took 95 days and 13 h.

## Results

### Patient Characteristics

We analyzed 12-lead ECG data from patients who underwent CA to treat IVA at the Ningbo First Hospital of Zhejiang University from March 2007 to September 2019. After the CA procedure, 2 (0.37%) of the patients developed a slight ecchymosis. All patients remained in the study, a total of 545, were randomly assigned to one of three cohorts, a training cohort (80%), which was used to train machine learning algorithms, a validation cohort (10%) that was designed to tune training parameters, and a testing cohort (10%) on which the machine learning algorithms were tested. The baseline characteristics of 545 patients are presented in Table 3 and a total of 18,612 ECG recordings were extracted from these patients. The ECG recording sample allocations of four classification schemes were presented in Table 2 and Supplementary Tables 1–3. There were no statistically significant differences with respect to age, male, body mass index, left ventricular ejection fraction, PVC, and clinical arrhythmia across the three groups.

### Assessing the Performance of the Models

After comparing over one hundred machine learning algorithms that were successfully implemented in a similar study (7, 23), our results showed that the Extremely Randomized Trees classifier attained the highest performance in all settings as evidenced by its accuracy, F1-scores, and 95% CIs. All performance measures reported below were based on a blind test of the testing cohorts that encompass ECG recordings associated with anatomic sites in each classification scheme.

In the first classification scheme used to predict right ventricular endocardium, left ventricular endocardium, and epicardium of left ventricular summit, the model achieved an accuracy of 99.79 (99.41–99.89) and an F1-score of 99.84 (99.6–99.96). For scheme 2, designed to predict 5 possible locations, the proposed method reached an accuracy of 99.62 (99.09–99.78) and an F1-score of 99.42 (98.79–99.75). For scheme 3 that predicts 18 anatomical locations, the model achieved an accuracy of 97.78 (96.76–98.41), an F1-score of 97.74 (94.15–99.73), and an adjusted accuracy of 98.53 (98.33–99.15). For scheme 4 that can distinguish 21 origin sites, the proposed model attained an accuracy of 98.24 (97.36–98.71), an F1-score of 98.56 (97.88–99.12), and an adjusted accuracy of 98.75 (98.35–99.38). These prediction accuracy performance measures and anatomical sites associated with each scheme were presented in Table 1. The performance measures for each sites in scheme 4 were shown in Table 4. Similar prediction performance reports for scheme 1–3 were presented in Supplementary Tables 4–6. Considering the general accuracy does not take into account the subtleties of class size imbalances, we reported F1-scores that represent the harmonic mean of the estimated precision and recall. Moreover, for classification schemes 3 and 4, we developed a new scoring metric that awards partial credit to incorrect predictions that resulted in similar treatments or outcomes as the true locations would have produced. We reported the new adjusted accuracy results using these new scoring metrics in Supplementary Tables 7, 8.

**Table 4:** Performance report with 95% CIs for classification scheme 4.

**Locations**	**SE (%)**	**SP (%)**	**PPV (%)**	**NPV (%)**	**F1–Score (%)**	**Balanced ACC (%)**
AC	98.15 (93.09–100)	99.78 (99.37–99.93)	97.25 (92.88–99.19)	99.85 (99.42–100)	97.7 (94.97–99.24)	98.96 (96.46–99.89)
AMC	98.61 (95.16–100)	99.92 (99.55–100)	99.3 (95.97–100)	99.85 (99.47–100)	98.95 (97.02–99.68)	99.27 (97.52–99.96)
RVOT posterior septal	100 (NA)	99.77 (99.38–99.92)	98.4 (95.75–99.49)	100 (NA)	99.2 (97.83–99.75)	99.88 (99.69–99.96)
Epicardium of LV summit	100 (NA)	100 (NA)	100 (NA)	100 (NA)	100 (NA)	100 (NA)
RVOT free wall	99.22 (95.68–100)	99.78 (99.40–99.93)	97.71 (93.92–99.32)	99.93 (99.62–100)	98.46 (96.34–99.61)	99.5 (97.73–99.93)
RVOT anterior septal	99.01 (94.40–100)	100 (NA)	100 (NA)	99.93 (99.57–100)	99.5 (97.12–100)	99.5 (97.20–100)
LC	97.17 (93.88–98.98)	99.6 (99.12–99.84)	97.63 (94.69–99.10)	99.53 (98.97–99.84)	97.4 (95.24–98.63)	98.39 (96.74–99.26)
LCC	100 (NA)	99.93 (99.58–100)	98.33 (91.18–100)	100 (NA)	99.16 (95.38–100)	99.96 (99.79–100)
LCC–RCC commissure	100 (NA)	100 (NA)	100 (NA)	100 (NA)	100 (NA)	100 (NA)
LPF	100 (NA)	99.71 (99.28–99.93)	95.79 (90.12–98.88)	100 (NA)	97.85 (94.80–99.44)	99.86 (99.64–99.96)
LPPM	98.53 (90.86–100)	100 (NA)	100 (NA)	99.93 (99.57–100)	99.26 (95.21–100)	99.26 (95.43–100)
LAF	95.51 (88.52–98.8)	99.93 (99.64–100)	98.84 (94.21–100)	99.71 (99.28–99.93)	97.14 (93.48–98.91)	97.72 (94.27–99.35)
LAPM	100 (NA)	100 (NA)	100 (NA)	100 (NA)	100 (NA)	100 (NA)
MV	93.94 (80–100)	100 (NA)	100 (NA)	99.86 (99.52–100)	96.88 (88.89–100)	96.97 (90–100)
Left His bundle	100 (NA)	100 (NA)	100 (NA)	100 (NA)	100 (NA)	100 (NA)
Right His bundle	94.37 (86.18–98.44)	99.86 (99.43–100)	97.1 (90.12–100)	99.72 (99.29–99.93)	95.71 (90.53–98.14)	97.11 (93.17–99.16)
RC	94.29 (81.31–100)	99.86 (99.51–100)	94.29 (80–100)	99.86 (99.49–100)	94.29 (85.71–98.51)	97.07 (90.71–99.93)
RCC	100 (NA)	100 (NA)	100 (NA)	100 (NA)	100 (NA)	100 (NA)
RAPM	100 (NA)	100 (NA)	100 (NA)	100 (NA)	100 (NA)	100 (NA)
Summit	100 (NA)	100 (NA)	100 (NA)	100 (NA)	100 (NA)	100 (NA)
TV	98.21 (93.19–99.16)	99.93 (99.17–100)	98.21 (96.37–99.96)	99.93 (99.26–100)	98.21 (95.47–99.46)	99.07 (98.33–100)
Average	98.43 (97.61–99.5)	99.9 (98.01–100)	98.71 (97.85–99.36)	98.43 (97.65–99.42)	98.56 (97.88–99.12)	98.24 (97.36–98.71)

SE, sensitivity; SP, specificity; PPV, positive predictive value; NPV, native predictive value; ACC, accuracy; F1-Score =2 * PPV * SE / (PPV + SE); Balanced ACC = (SE+SP)/2; NA, not applicable; other abbreviations are as in Table 1.

**Figure 6:**
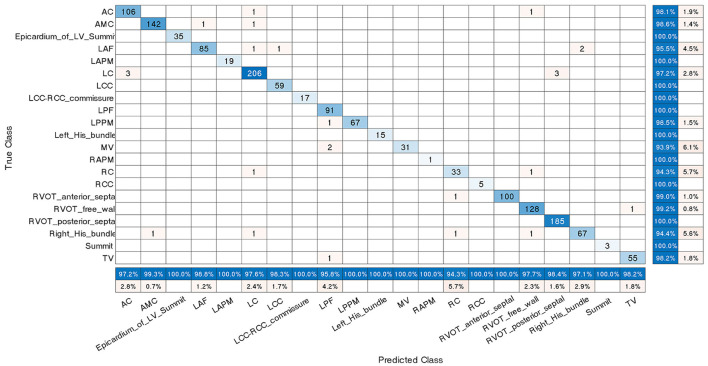
Confusion matrix for classification scheme 4. The true class labels of the 21 origins in the *Y*-axis were confirmed by successful CA. The predicted class labels in the *X*-axis represent the outcomes generated by the machine learning classification model. Numbers in blue on the main diagonal represent the correct predictions. Percentages in blue represent the accuracy of the corresponding category. A number presented in matrix is the amount of ECG recording samples associated with an anatomic label, not the count of patients. The abbreviations are as in Table 1.

Furthermore, the confusion matrix shown in Figure 6 describes the performance of the classification scheme 4. It presents both correct and incorrect frequency counts for the model predictions of each site compared against the true locations. The numbers presented in matrix are the amount of ECG recording samples, not the count of patients. Similar confusion matrices for the other three classification schemes were presented in Supplementary Figures 1–3.

### Presentation and Interpretation of the Fitted Models

**Figure 7:**
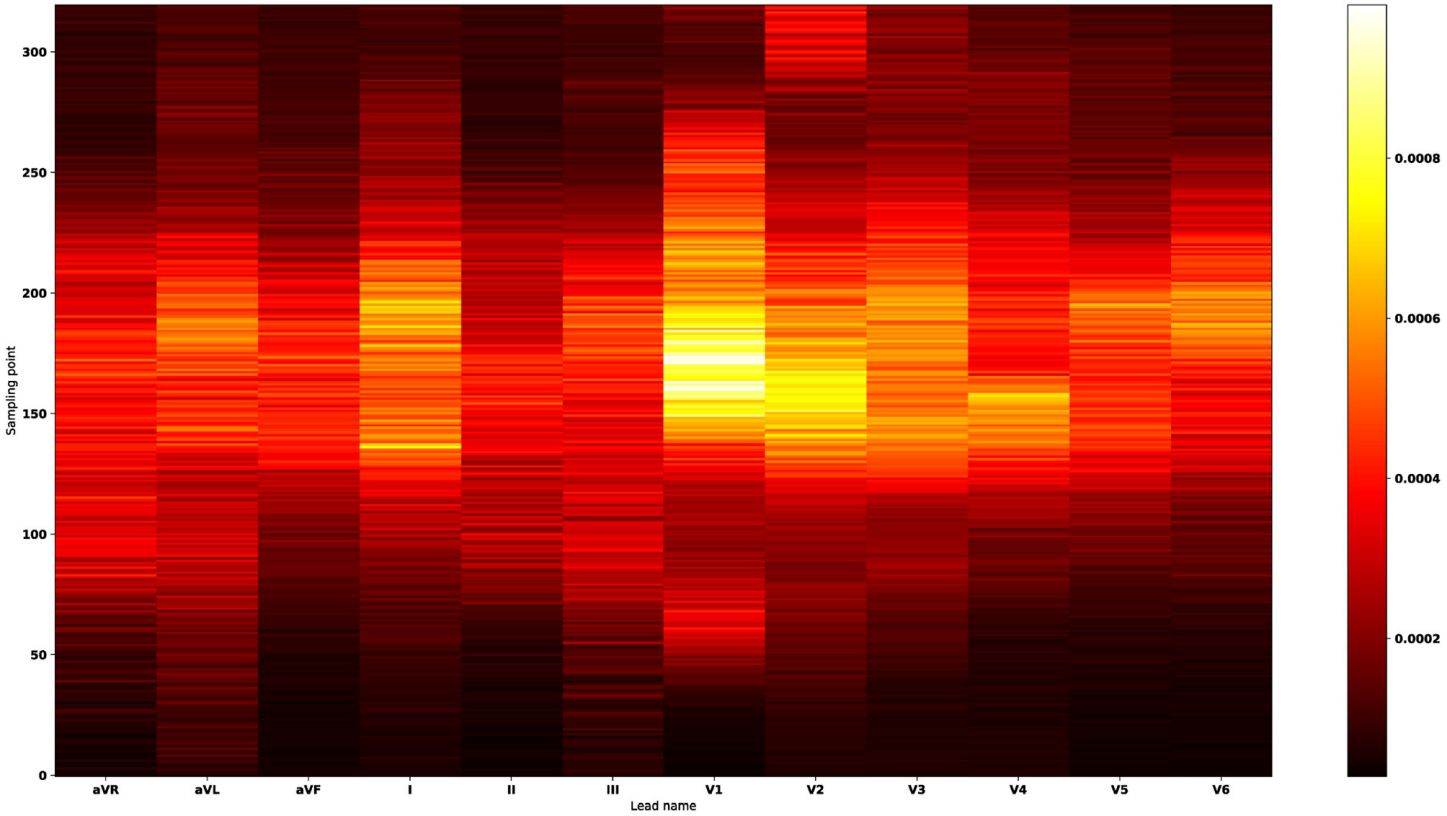
Feature importance heat map for classification scheme 4. The *X*-axis denotes 12 leads, and the *Y*-axis indicates the sampling point. The ascending color brightness indicates the increasing variable importance magnitudes. The most important features occur in the lead-V1, lead-V2, lead-I, lead-V3, lead-V4, lead-V5, and lead-V6 sequentially. Thus, the feature importance heat map suggests that lead-V1 plays a major role in predicting the 21 anatomical origins. By ranking the signals according to the magnitudes of variable importance, we found the top three important features: in lead-V1 the voltage value at the 10th point after the reference line; in lead-V1 the voltage value at the reference line; in lead-V1 the voltage value at the 3rd point after the reference line. The point in the reference line (the R-wave peak point at lead-II) is counted as the first point.

In machine learning, the feature importance is used to measure the magnitude of variable impact in predicting the site of origin. By ranking the features according to the magnitude of the impact they have on predicting the site of origin, we created a new feature importance heat map (shown in Figure 7) to visualize the variable importance for classification scheme 4. The corresponding feature importance heat maps for classification schemes 1–3 were presented in the Supplementary Figures 4–6. The feature importance heat map allows us to identify the top three important features and utilize them to make preliminary origins estimation. For instance, we have found that the voltage value at the 10th position in lead V1 after the reference line is the variable with the highest importance. Through further single variable analysis (shown in Supplementary Figure 14), one can find that the average voltage value of this variable for aortomitral continuity is higher than the average value for right coronary cusp. Also, this average value is lower for the tricuspid valve location compared to all other origins in the left ventricular endocardium. The voltage value at the 7th position in lead-V1 after the reference line is the most important variable to separate left ventricular endocardium, right ventricular endocardium, and epicardium of left ventricular summit (shown in Supplementary Figure 9). The high average value of this variable was associated with a high probability that the location is on the left ventricular endocardium. Similarly, one can utilize the other important variables presented in Supplementary Figures 7–9 to precisely determine the misfiring locations.

**Figure 8:**
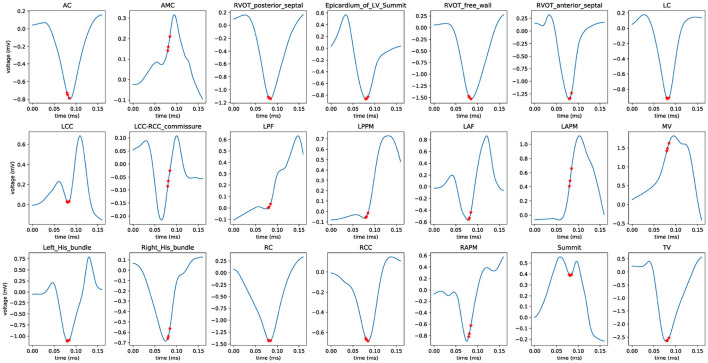
Average ECG morphology of V1 lead for classification scheme 4. Nonparametric smoothing technique was employed to generate the average V1-lead ECG morphology for all 21 anatomical sites. The three red stars denote the locations of the three most important variables (ECG signals). The abbreviations used are identical to those in Table 1.

Furthermore, we employed a non-parametric smoothing technique to visualize the average ECG morphology and important signal locations. For example, a total of 863 V1-lead ECG recordings that have the label of AC site in the scheme 4 training cohort were smoothed and averaged to a single line (shown in subplot entitled with AC in Figure 8). The average V1-lead ECG morphologies for the remaining 20 anatomical sites were shown in the same figure.

### Failure Prediction Analysis

The proposed machine learning model will report the probabilities of all possible sites and their corresponding rankings. For instance, in classification scheme 4 the algorithm's exceptional accuracy means that in almost all cases the machine learning model ranks the correct site as number one (1,451 out of 1,476). However, if the top prediction is incorrect, the location with the second highest probability is likely the correct site. We considered 26 predictions with incorrect machine learning model top predictions. In 20 of these cases, the second ranked location identified the true site and in 6 of these cases the third ranked location did too. The probabilities of all possible sites for these 26 samples were presented in Supplementary Table 11.

Moreover, we provide visualization results from model output and input of failed predictions. For instance, to analyze a sample in the testing cohort that was predicted as an AC site but the true class is RVOT anterior septal, three plots were presented in Supplementary Figure 15 consisting of the average 12-lead ECG graphic with true label, a 12-lead ECG data in testing cohort that was misclassified, and the average 12-lead ECG data with the false prediction label.

### Comparison With Previous Studies and Human Experts

We compared our approach against results from 15 prior studies (8–16, 18–22, 29) as well as prediction performance of human experts. The accuracy, F1-Score, sensitivity, specificity, positive predictive value, negative predictive value, and the area under the curve of the receiver operating characteristic (ROC) curve were used to compare performances and were shown in Supplementary Table 9. The proposed machine learning approach attained the highest overall prediction accuracy (0.9824) and achieved the best anatomical precision by identifying the largest number of sites (21).

We also designed a comprehensive study to compare the location prediction performance between human experts and our machine learning algorithm. A total of 12 ablation operators who had 5 to 20 years of radiofrequency catheter ablation experience carried out ablation procedures to 545 patients. Under the multi-class setting, we used one vs. others strategy to compute sensitivity, specificity, F1-score, and accuracy for each site. For instance, if the successful ablation site is AC and the first ablation site given by an operator was AC, this was counted as a true positive for the AC site. If the successful ablation site was AC and the first ablation site given by operator was not AC, this was counted as a false negative for the AC site. If the successful ablation site was not AC and the first ablation site given by operator was AC, this was counted as a false positive for the AC site. If the successful ablation site was not AC and the first ablation site given by operator was not AC, this was counted as a true negative for the AC site. Sequentially, after we calculated these measurements for all 21 sites, reported the weighted averages of these performance metrics (shown in Supplementary Table 10). In summary, the sensitivity, specificity, F1-Score, and accuracy of the machine learning-enabled ECG approach exceeded those of the human experts 0.57, 18.18, 2.17, and 3.95%, respectively.

## Discussion

Accurate prediction of the source of IVA is challenging and can be further obfuscated by the presence of additional abnormal findings on the ECG, such as a bundle block. Even though mapping can easily determine the sites, it is disadvantageous to map every possible site during the ablation procedure. The additional and redundant mapping, especially for the cross left and right chamber one, will prolong the operation time and increase the risk of complications. For example, some origins including AMC, epicardium of LV summit, LVOT and RVOT, have very similar ECG morphology. In these scenarios, if the sites can be accurately predicted, the extra mapping for the remaining possible ablation sites is not necessary and can be avoided. Therefore, developing and deploying an algorithm with high prediction accuracy to guide the practitioners in identifying the likely target of ablation would be invaluable. The assistive technology will improve success rates, enhance and accelerate the procedure planning, and enable detailed discussion of the treatment options and risks with the patients.

We have collected a large expertly labeled database consisting of 18,612 ECG recordings recorded from 545 patients, we trained and validated a state-of-the-art machine learning algorithm to predict the exact 21 anatomical origins of IVA with an accuracy of 98.24%. Under the auspices of Chapman University and Ningbo First Hospital of Zhejiang University, we created and shared our proprietary 12-lead ECG database interpreted by experts with the scientific community (30).

A comprehensive comparison of the predictive accuracy of our approach against methods proposed in 15 previous studies (8–16, 18–22, 29) showed that our algorithm is the only one that can predict the largest number of sites (21) with an accuracy of 98.24%. None of the existing research results managed to achieve prediction of the IVA sites of origin with the anatomical level of detail of our study. The machine learning algorithm makes a prediction based on the complete data consisting of all available smoothed voltage values while previous studies used only a set of variables extracted from the raw data, such as the transitional zone index and R-wave amplitudes. For classification scheme 4, designed to predict 21 possible anatomical sites, the univariate and trivariate analysis demonstrated that neither single nor triple variable combination could separate all possible origins clearly (Supplementary Figures 14, 16).

Moreover, this algorithm was designed to support ablation operator without breaking current ablation practices. For instance, if PVC or VT originated from one site with multiple PVC exit sites (which presented multiple QRS morphologies), the operator would choose QRS morphology of the most frequent PVC to predict the possible ablation site and deploy the ablation procedure introduced in the section of Catheter ablation procedures. Finally, the acute ablation success will confirm whether such an assumption is true. This algorithm will take ECG data as input (decided by operator) and predict the possible origin site. Thus, it will seamlessly help operator to make the correct decision and improve the probability of success.

### Study Limitations

Although this study includes patients with a comprehensive list of anatomy sites, the oversampling was carried out during the training stage to balance minority representations of RCC and summit under LVOT, left His bundle and RAPM, and the performance of the method could be improved in the presence of more cases of them. As this retrospective study spanned a long period of time and intracardiac echo was not utilized in all cases (especially at early stages of the study), some exact locations were not strictly ascertained by supporting evidence from intracardiac echo, such as pulmonary cusps and papillary muscle origins. Moreover, some cases of multi-ablation at different sites for multi-focal PVC/VT were excluded from the study. Thus, the algorithm potentially could have a limitation if applied in such scenarios. A multi-center prospective evaluation in larger cohorts is necessary to show robustness and compatibility of the proposed algorithm.

## Conclusion

The proposed machine learning model can be immediately and effortlessly deployed to electrophysiology labs allowing cardiologists to predict the exact origins of arrhythmia and provide an optimum treatment plan both before and during the CA procedure. The complete analysis was done automatically by the model, and the prediction for one patient only takes less than one second of computational time.

## Data Availability Statement

The ECG data, analytical methods, and source code are available in Figshare https://doi.org/10.6084/m9.figshare.c.4668086.v2 and the MATALB program 384 for ECG denoising is put under https://github.com/zheng120/PVCVTECGDenoising.

## Ethics Statement

The studies involving human participants were reviewed and approved by the Institutional Review Board of Ningbo First Hospital of Zhejiang University. The Ethics Committee waived the requirement of written informed consent for participation.

## Author Contributions

JZ, GF, IA, TC, HC, and CR: processed the data for analysis. JZ, HC, KA, GF, and CR: performed the statistical analysis. All authors have made a substantial, direct and intellectual contribution to the study design, data interpretation, and writing of the report.

## Funding

This work was supported by the 2020 Natural Science Foundation of Zhengjiang Province (ID LGJ20H020001) to HC.

## Conflict of Interest

JZ and HC are the inventors of intellectual property in the field of ventricular arrhythmia origins identification methods. HC has served as a consultant for Biosense Webster, Boston Scientific, and Abbott. The remaining authors declare that the research was conducted in the absence of any commercial or financial relationships that could be construed as a potential conflict of interest.

## Publisher's Note

All claims expressed in this article are solely those of the authors and do not necessarily represent those of their affiliated organizations, or those of the publisher, the editors and the reviewers. Any product that may be evaluated in this article, or claim that may be made by its manufacturer, is not guaranteed or endorsed by the publisher.
