# Supplementary material for: A High Precision Machine Learning-Enabled System for Predicting Idiopathic Ventricular Arrhythmia Origins
Source: Front Cardiovasc Med. 2022 Mar 11;9:809027. doi: 10.3389/fcvm.2022.809027 (PMC8962834; doi:10.3389/fcvm.2022.809027)
Supplement: Supplementary file 1 [file Data_Sheet_1.PDF]

A High Precision Machine Learning-enabled System for Predicting Idiopathic Ventricular Arrhythmia Origins

Jianwei Zheng, Ph.D.[1], Guohua Fu M.D.[2], Daniele Struppa, Ph.D.[1], Islam Abudayyeh, M.D., MPH.[3], Tahmeed Contractor, M.D.[3], Xianfeng Du, M.D.[2], Kyle Anderson, Ph.D.[1], Huimin Chu MD.*[2], and Cyril Rakovski, Ph.D.[1]

[1]Chapman University
[2]Ningbo First Hospital of Zhejiang University
[3]Loma Linda University Health

Supplementary Material

*Corresponding author, Address: 59 Liuting St, Haishu, Ningbo, Zhejiang, China, 315016.
Email: mark.chuhuimin@gmail.com

Table 1: Classification Scheme 1: RV = Right ventricle; LV = Left ventricle

Locations	Patients, n (%)	ECG Recordings, n (%)	Training cohort,n	Validation cohort,n	Testing cohort,n
RV endocardium	374 (68.63)	11,917 (64.02)	9,533	1,192	1,192
LV endocardium	161 (29.54)	6,344 (34.9)	5,075	634	635
Epicardium of LV summit	10 (1.83)	351 (1.89)	281	35	35

Table 2: Classification Scheme 2: LV = Left ventricle

	Patients, n (%)	ECG Recordings, n (%)	Training cohort,n	Validation cohort,n	Testing cohort,n
Outflow tract endocardium	414 (75.96)	13,398 (71.99)	10,718	1,340	1,340
Non-outflow tract endocardium	121 (22.2)	4,863 (26.12)	3,889	487	487
Epicardium of LV summit	10 (1.84)	351 (1.89)	281	35	35

Table 3: Classification Scheme 3: RVOT = Right ventricular outflow tract; LVOT = Left ventricular outflow tract; RVNOT = Right ventricular non-outflow tract; LVNOT = Left ventricular non-outflow tract; LV = Left ventricle

Locations	Patients, n (%)	ECG Recordings, n (%)	Training cohort	Validation cohort	Testing cohort
RVOT endocardium	322(59.08)	10,625(57.09)	8,501	1,062	1,062
LVOT endocardium	92(16.88)	2,773(14.9)	2,217	278	278
LVNOT	69(12.66)	3,571(19.19)	2,857	357	357
RVNOT	52(9.54)	1,292(6.94)	1,034	129	129
Epicardium of LV summit	10(1.83)	351(1.89)	281	35	35

Table 4: Classification Scheme 4: LC = left cusp; LCC = left coronary cusp; AC = anterior cusp; RC = right cusp; AMC = aortomitral continuity; MV= mitral valve; TV = tricuspid valve; RCC = right coronary cusp; RAPM = right anterior papillary muscle; LV = Left ventricle

Locations	Patients, n (%)	ECG Recordings, n (%)	Training cohort	Validation cohort	Testing cohort
Right septal	134(25.28)	4,686(26.68)	3,748	469	469
LC	82(15.47)	2,664(15.17)	2,130	267	267
LCC	42(7.92)	590(3.36)	472	59	59
Left septal	40(7.55)	2,220(12.64)	1,776	222	222
AC	40(7.55)	1,091(6.21)	873	109	109
RVOT free wall	32(6.04)	1,287(7.33)	1,029	129	129
Right His bundle	31(5.85)	705(4.01)	563	71	71
RC	24(4.53)	353(2.01)	283	35	35
AMC	23(4.34)	1,434(8.16)	1,146	144	144

Table 4 continued from previous page

Locations	Patients, n (%)	ECG Recordings, n (%)	Training cohort	Validation cohort	Testing cohort
TV	18(3.4)	563(3.21)	451	56	56
Left papillary muscle	18(3.4)	872(4.96)	698	87	87
LCC-RCC commisure	11(2.08)	172(0.98)	138	17	17
Epicardium of LV summit	10(1.89)	351(2)	281	35	35
MV	8(1.51)	330(1.88)	264	33	33
RCC	7(1.32)	51(0.29)	41	5	5
Summit	5(0.94)	31(0.18)	25	3	3
Left His bundle	3(0.57)	149(0.85)	119	15	15
RAPM	2(0.38)	14(0.08)	12	1	1

Table 5: Classification scheme 5 for 21 sites of origin. LC = left cusp; LCC = left coronary cusp; AC = anterior cusp; RC = right cusp; AMC = aortomitral continuity; LPF = left posterior fascicle; TV = tricuspid valve; LAF = left anterior fascicle; LPPM = left posterior papillary muscle; MV= mitral valve; LAPM = left anterior papillary muscle; RCC = right coronary cusp; RAPM = right anterior papillary muscle.

Locations	Patients, n(%)	ECG Recordings, n(%)	Training cohort, n(%)	Validation cohort, n(%)	Testing cohort, n(%)
LC	67(15.12)	2,118(14.36)	1,694(80)	212(10)	212(10)
RVOT posterior septal	43(9.93)	1,848(12.53)	1,478(80)	185(10)	185(10)
LCC	41(9.47)	588(3.99)	470(80)	59(10)	59(10)
AC	38(8.78)	1,079(7.31)	863(80)	108(10)	108(10)
RVOT free wall	32(7.39)	1,287(8.72)	1,029(80)	129(10)	129(10)
RVOT anterior septal	32(7.39)	1,014(6.87)	812(80)	101(10)	101(10)
Right His bundle	31(7.16)	705(4.78)	563(80)	71(10)	71(10)
RC	24(5.54)	353(2.39)	283(80)	35(10)	35(10)
AMC	23(5.31)	1,434(9.72)	1,146(80)	144(10)	144(10)
LPF	18(4.16)	913(6.19)	731(80)	91(10)	91(10)
TV	18(4.16)	563(3.82)	451(80)	56(10)	56(10)
LAF	13(3)	885(6)	707(80)	89(10)	89(10)
LCC-RCC commissure	11(2.54)	172(1.17)	138(80)	17(10)	17(10)
Epicardium of LV summit	10(2.31)	351(2.38)	281(80)	35(10)	35(10)
LPPM	9(2.08)	677(4.59)	541(80)	68(10)	68(10)
MV	8(1.85)	330(2.24)	264(80)	33(10)	33(10)
LAPM	8(1.85)	192(1.3)	154(80)	19(10)	19(10)
RCC	7(1.62)	51(0.35)	41(80)	5(10)	5(10)
Summit	5(1.15)	31(0.21)	25(80)	3(10)	3(10)
Left His bundle	3(0.69)	149(1.01)	119(80)	15(10)	15(10)
RAPM	2(0.46)	14(0.09)	12(80)	1(10)	1(10)

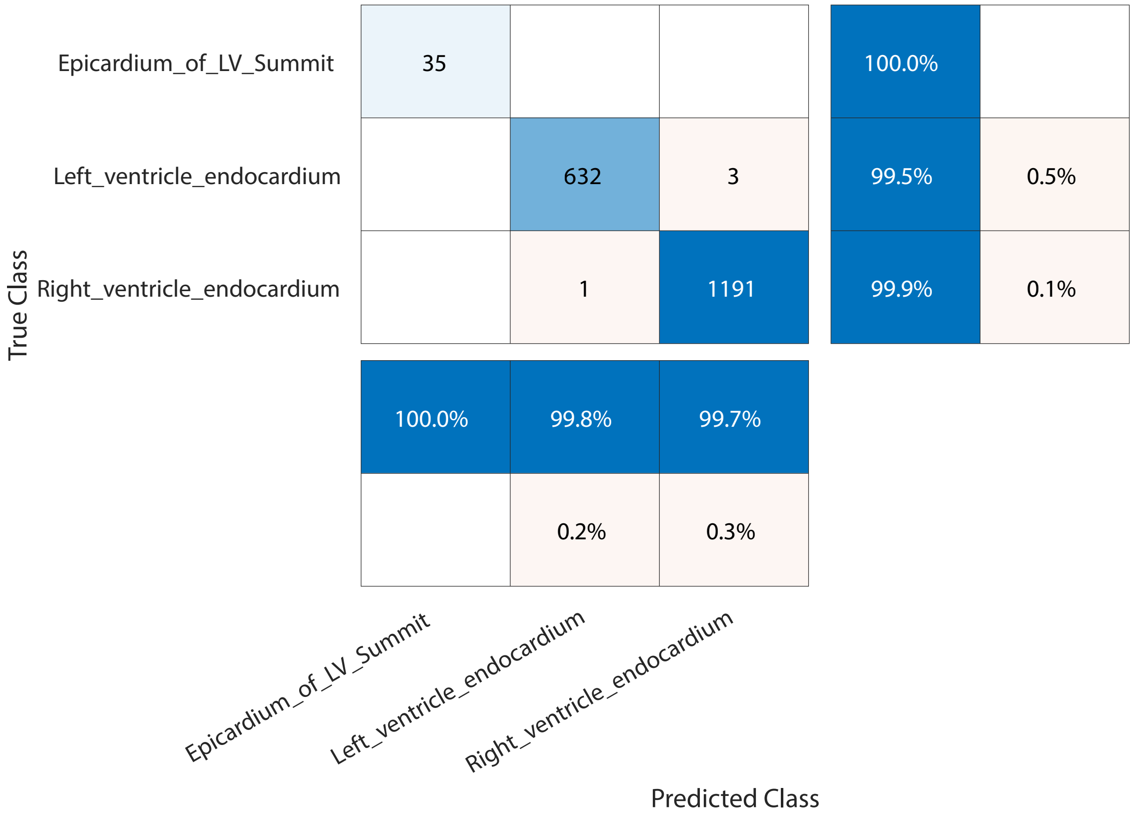

Figure 1: Confusion matrix for classification scheme 1. The true location labels of the 3 origins were confirmed by successful CA. The predicted location labels represent the outcomes generated by classification model. Numbers in blue on the main diagonal represent the correct predictions. Percentages in blue represent the accuracy for the corresponding category.

Table 6: Performance report with 95% CIs for classification scheme 1. SE = sensitivity; SP = specificity; PPV = positive predictive value; NPV = native predictive value; ACC = accuracy; F1-Score =2 * PPV * SE / (PPV + SE); Balanced ACC = (SE+SP)/2; NA = not applicable; LV = Left ventricle; RV = Right ventricle.

Locations	SE(%)	SP(%)	PPV(%)	NPV(%)	F1-Score(%)	Balanced ACC(%)
Epicardium of LV summit	100 (NA)	100 (NA)	100 (NA)	100 (NA)	100 (NA)	100 (NA)
LV endo-cardium	99.53 (98.60-99.85)	99.92 (99.51-100)	99.84 (99.06-100)	99.76 (99.28-99.92)	99.68 (99.21-99.92)	99.72 (99.27-99.92)
RV endo-cardium	99.92 (99.50-100)	99.55 (98.67-99.86)	99.75 (99.25-99.92)	99.85 (99.11-100.00)	99.83 (99.58-99.96)	99.73 (99.30-99.93)

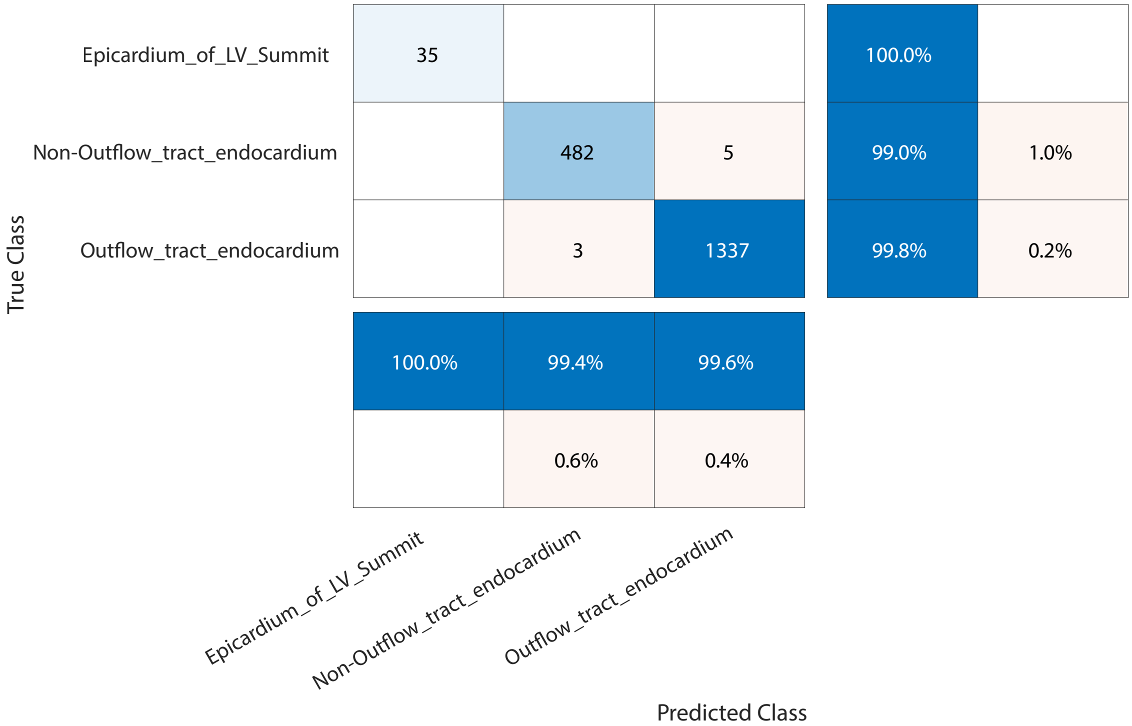

Figure 2: Confusion matrix for classification scheme 2. The true location labels of the 3 origins were confirmed by successful CA. The predicted location labels represent the outcomes generated by the classification model. Numbers in blue on the main diagonal represent the correct predictions. Percentages in blue represent the accuracy for the corresponding category.

Table 7: Performance report with 95% CIs for classification scheme 2. The abbreviations are as in Table 1 and 5.

Locations	SE(%)	SP(%)	PPV(%)	NPV(%)	F1-Score(%)	Balanced ACC(%)
Epicardium of LV summit	100 (NA)	100 (NA)	100 (NA)	100 (NA)	100 (NA)	100 (NA)

Table 7 continued from previous page

Locations	SE(%)	SP(%)	PPV(%)	NPV(%)	F1-Score(%)	Balanced ACC(%)
Non-Outflow tract endocardium	98.97 (97.68-99.60)	99.78 (99.41-99.93)	99.38 (98.27-99.81)	99.64 (99.19-99.86)	99.18 (98.46-99.61)	99.38 (98.73-99.73)
Outflow tract endocardium	99.78 (99.40-99.93)	99.04 (97.83-99.63)	99.63 (99.17-99.85)	99.42 (98.39-99.82)	99.7 (99.44-99.85)	99.41 (98.79-99.74)

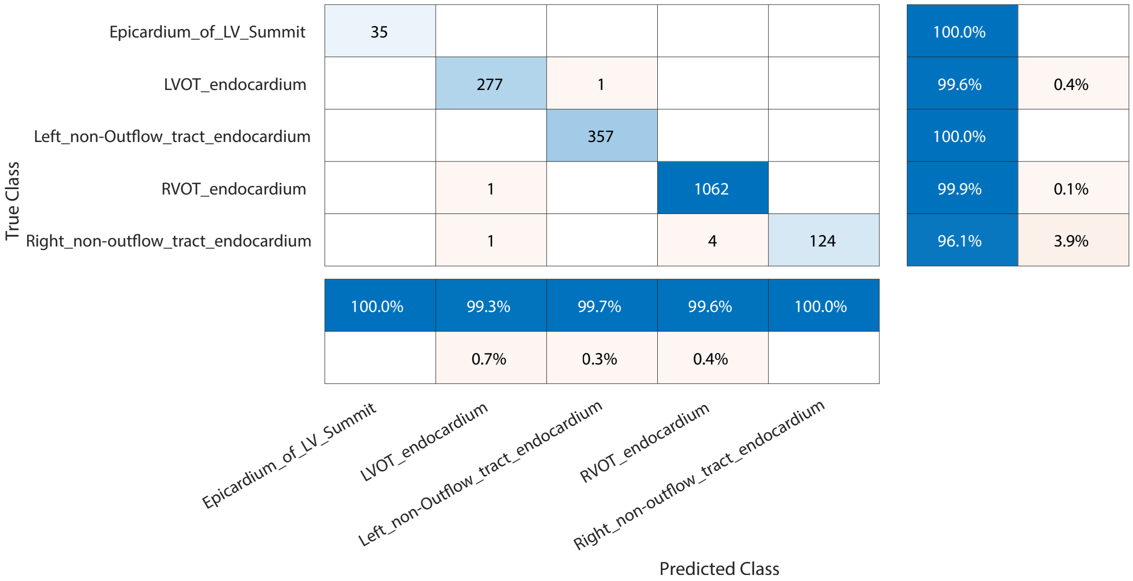

Figure 3: Confusion matrix for classification scheme 3. The true location labels of the 5 origins were confirmed by successful CA. The predicted location labels represent the outcomes generated by the classification model. Numbers in blue on the main diagonal represent the correct predictions. Percentages in blue represent the accuracy for the corresponding category.

Table 8: Performance report with 95% CIs for classification scheme 3. The abbreviations are as in Table 3 and 5.

Locations	SE(%)	SP(%)	PPV(%)	NPV(%)	F1-Score(%)	Balanced ACC(%)
Epicardium of LV summit	100 (NA)	100 (NA)	100 (NA)	100 (NA)	100 (NA)	100 (NA)
Left non-outflow tract endocardium	100 (NA)	99.93 (99.61-100)	99.72 (98.27-100)	100 (NA)	99.86 (99.13-100)	99.97 (99.80-100)
LVOT endocardium	99.64 (97.95-100)	99.87 (99.56-100)	99.28 (97.50-100)	99.94 (99.62-100)	99.46 (98.54-99.83)	99.76 (98.94-99.97)
Right non-outflow tract endocardium	96.12 (91.77-98.56)	100 (NA)	100 (NA)	99.71 (99.36-99.89)	98.02 (95.71-99.28)	98.06 (95.88-99.28)
RVOT endocardium	99.91 (99.52-100)	99.5 (98.81-99.87)	99.62 (99.10-99.91)	99.87 (99.37-100)	99.77 (99.48-99.91)	99.7 (99.36-99.89)

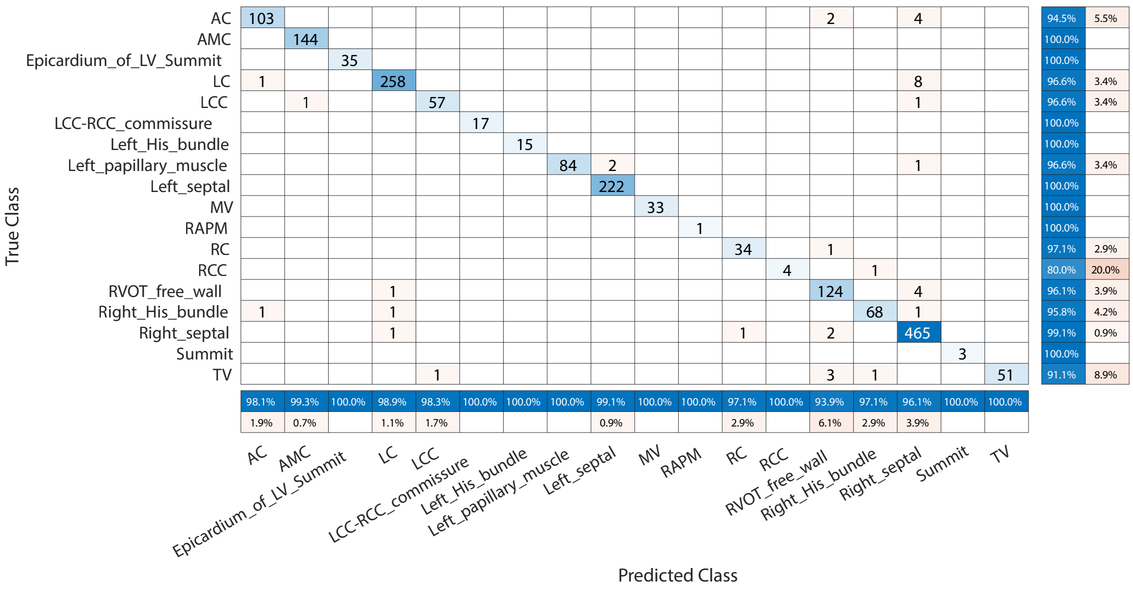

Figure 4: Confusion matrix for classification scheme 4. The true location labels of the 18 origins were confirmed by successful CA. The predicted location labels represent the outcomes generated by the classification model. Numbers in blue on the main diagonal represent the correct predictions. Percentages in blue represent the accuracy for the corresponding category. LCC = left coronary cusp; RCC = right coronary cusp; AMC = aortomitral continuity; MV= mitral valve; AC = anterior cusp; LC = left cusp; RC = right cusp; TV = tricuspid valve; RAPM = right anterior papillary muscle.

Table 9: Performance report with 95% CIs for classification scheme 4. LCC = left coronary cusp; RCC = right coronary cusp; AMC = aortomitral continuity; MV= mitral valve; AC = anterior cusp; LC = left cusp; RC = right cusp; TV = tricuspid valve; RAPM = right anterior papillary muscle; NA = not applicable. The other abbreviations are as in Table 4 and 5.

Locations	SE(%)	SP(%)	PPV(%)	NPV(%)	F1-Score(%)	Balanced ACC(%)
AC	94.5 (88.90-98.00)	99.88 (99.57-100)	98.1 (93.62-100)	99.64 (99.22-99.88)	96.26 (92.89-98.23)	97.19 (94.40-98.93)
AMC	100 (NA)	99.94 (95.98-100)	99.31 (97.95-100)	100 (NA)	99.65 (91.47-98.54)	99.97 (99.03-99.76)
Epicardium of LV summit	100 (NA)	100 (NA)	100 (NA)	100 (NA)	100 (NA)	100 (NA)
RVOT free wall	96.12 (88.52-97.08)	99.51 (99.32-99.88)	93.94 (91.47-97.28)	99.69 (95.51-99.06)	95.02 (93.70-98.35)	97.82 (96.40-99.93)
LC	96.63 (96.31-99.64)	99.8 (99.58-100)	98.85 (97.5-100)	99.4 (98.98-99.8)	97.73 (95.58-99.02)	98.21 (94.1-99.97)
LCC	96.61 (89.92-98.98)	99.94 (99.46-100)	98.28 (94.70-99.49)	99.88 (99.54-100)	97.44 (96.83-100)	98.28 (96.39-100)
LCC-RCC commissure	100 (NA)	100 (NA)	100 (NA)	100 (NA)	100 (NA)	100 (NA)
Left papillary muscle	96.55 (93.70-98.35)	100 (NA)	100 (NA)	99.82 (98.39-100)	98.25 (96.31-100)	98.28 (97.09-100)

Table 9 continued from previous page

Locations	SE(%)	SP(%)	PPV(%)	NPV(%)	F1-Score(%)	Balanced ACC(%)
Left septal	100 (NA)	99.87 (92.15-100)	99.11 (98.39-100)	100 (NA)	99.55 (94.96-99.49)	99.93 (94.77-99.49)
MV	100 (NA)	100 (NA)	100 (NA)	100 (NA)	100 (NA)	100 (NA)
Left His bundle	100 (NA)	100 (NA)	100 (NA)	100 (NA)	100 (NA)	100 (NA)
Right His bundle	95.77 (88.17-98.75)	99.88 (99.58-100)	97.14 (90.41-100)	99.82 (99.47-99.94)	96.45 (92.14-98.69)	97.83 (94.15-99.33)
RC	97.14 (82.19-100)	99.94 (99.65-100)	97.14 (83.23-100)	99.94 (99.65-100)	97.14 (88.46-100)	98.54 (91.56-100)
RCC	80 (76-91.22)	100 (NA)	100 (NA)	99.94 (99.58-100)	88.89 (88.04-100)	90 (89.92-98.98)
RAPM	100 (NA)	100 (NA)	100 (NA)	100 (NA)	100 (NA)	100 (NA)
RightSeptal	99.15 (98.18-99.73)	98.52 (96.88-99.93)	96.07 (93.33-99.13)	99.69 (99.18-100)	97.59 (95.66-99.39)	98.84 (96.48-99.88)
Summit	100 (NA)	100 (NA)	100 (NA)	100 (NA)	100 (NA)	100 (NA)
TV	91.07 (86.54-93.26)	100 (NA)	100 (NA)	99.71 (99.38-100)	95.33 (91.5-97.78)	95.54 (90.25-98.71)

Table 10: Score metric for classification scheme 4. The adjusted accuracy score awards partial credit to minor misprediction that results in similar treatments or outcomes as the true diagnosis as judged by CA outcomes. Let C = [c_i] be a collection of predictions. We compute a multi-class confusion matrix $A = a_{i,j}$, where $a_{i,j}$ is the number of recordings that were classified as belonging to class c_i but actually belong to class c_j. We assign different weights $W = w_{i,j}$ to different entries in this matrix based on the similarity of treatments or differences in CA procedure. The score S is given by $S = \sum_{i,j} a_{i,j} w_{i,j}$, which is an adjusted version of the traditional accuracy metric. 0 = AC; 1 = AMC; 2 = Epicardium of LV summit; 3 = RVOT free wall ; 4 = LC; 5 = LCC; 6 = LCC-RCC commissure; 7 = Left papillary muscle; 8 = Left septal; 9 = MV; 10 = Left His bundle; 11 = Right His bundle; 12 = RC; 13 = RCC; 14 = RAPM; 15 = Right septal; 16 = Summit; 17 = TV. The abbreviations are as in Table 4.

	0	1	2	3	4	5	6	7	8	9	10	11	12	13	14	15	16	17
0	1	0.2	0	0.4	0.5	0.2	0.2	0.1	0.1	0.1	0	0	0.5	0.2	0.3	0.3	0.2	0.3
1	0.2	1	0	0.2	0.2	0.5	0.5	0.3	0.3	0.3	0	0	0.2	0.5	0.1	0.2	0.4	0.1
2	0	0	1	0	0	0	0	0	0	0	0	0	0	0	0	0	0	0
3	0.4	0.2	0	1	0.4	0.2	0.2	0.1	0.1	0.1	0	0	0.4	0.2	0.3	0.5	0.2	0.3
4	0.5	0.2	0	0.4	1	0.2	0.2	0.1	0.1	0.1	0	0	0.5	0.2	0.3	0.4	0.2	0.3
5	0.2	0.5	0	0.2	0.2	1	0.5	0.3	0.3	0.3	0	0	0.2	0.5	0.1	0.2	0.4	0.1
6	0.2	0.5	0	0.2	0.2	0.5	1	0.3	0.3	0.3	0	0	0.2	0.5	0.1	0.2	0.4	0.1
7	0.1	0.3	0	0.1	0.1	0.3	0.3	1	0.5	0.4	0	0	0.1	0.3	0.1	0.1	0.2	0.1
8	0.1	0.3	0	0.1	0.1	0.3	0.3	0.5	1	0.4	0	0	0.1	0.3	0.1	0.1	0.2	0.1
9	0.1	0.3	0	0.1	0.1	0.3	0.3	0.4	0.4	1	0	0	0.1	0.3	0.1	0.1	0.2	0.1
10	0	0	0	0	0	0	0	0	0	0	1	0	0	0	0	0	0	0
11	0	0	0	0	0	0	0	0	0	0	0	1	0	0	0	0	0	0
12	0.5	0.2	0	0.4	0.5	0.2	0.2	0.1	0.1	0.1	0	0	1	0.2	0.3	0.4	0.2	0.3
13	0.2	0.5	0	0.2	0.2	0.5	0.5	0.3	0.3	0.3	0	0	0.2	1	0.1	0.2	0.4	0.1
14	0.3	0.1	0	0.3	0.3	0.1	0.1	0.1	0.1	0.1	0	0	0.3	0.1	1	0.3	0.1	0.2
15	0.3	0.2	0	0.5	0.4	0.2	0.2	0.1	0.1	0.1	0	0	0.4	0.2	0.3	1	0.1	0.2
16	0.2	0.4	0	0.2	0.2	0.4	0.4	0.2	0.2	0.2	0	0	0.2	0.4	0.1	0.1	1	0.1
17	0.3	0.1	0	0.3	0.3	0.1	0.1	0.1	0.1	0.1	0	0	0.3	0.1	0.2	0.2	0.1	1

Table 11: Score metric for classification scheme five. The adjusted accuracy score awards partial credit to minor misprediction that results in similar treatments or outcomes as the true diagnosis as judged by CA outcomes. Let C = [c_i] be a collection of predictions. We compute a multi-class confusion matrix $A = a_{i,j}$, where $a_{i,j}$ is the number of recordings that were classified as belonging to class c_i but actually belong to class c_j. We assign different weights $W = w_{i,j}$ to different entries in this matrix based on the similarity of treatments or differences in CA procedure. The score S is given by $S = \sum_{i,j} a_{i,j} w_{i,j}$, which is an adjusted version of the traditional accuracy metric. 0 = AC; 1=AMC; 2 = RVOT posterior septal; 3 = Epicardium of LV summit; 4 = RVOT free wall; 5 = RVOT anterior septal; 6 = LC; 7 = LCC; 8 = LCC-RCC commissure; 9 = Left posterior fascicle (LPF); 10 = Left posterior papillary muscle (LPPM); 11 = Left anterior fascicle (LAF); 12 = Left anterior papillary muscle (LAPM); 13 = MV; 14 = Left His bundle; 15 = Right His bundle; 16 = RC; 17 = RCC; 18 = Right anterior papillary muscle (RAPM); 19 = Summit; 20 = TV. The abbreviations are as in Table 4.

	0	1	2	3	4	5	6	7	8	9	10	11	12	13	14	15	16	17	18	19	20
0	1	0.2	0.4	0	0.4	0.4	0.5	0.2	0.2	0.1	0.1	0.1	0.1	0.1	0	0	0.5	0.2	0.3	0	0.3
1	0.2	1	0.2	0	0.2	0.2	0.2	0.5	0.5	0.3	0.3	0.3	0.3	0.3	0	0	0.2	0.5	0.1	0.4	0.1
2	0.4	0.2	1	0	0.5	0.5	0.4	0.2	0.2	0.1	0.1	0.1	0.1	0.1	0	0	0.4	0.2	0.3	0.2	0.3
3	0	0	0	1	0	0	0	0	0	0	0	0	0	0	0	0	0	0	0	0	0
4	0.4	0.2	0.5	0	1	0.5	0.4	0.2	0.2	0.1	0.1	0.1	0.1	0.1	0	0	0.4	0.2	0.3	0.2	0.3
5	0.4	0.2	0.5	0	0.5	1	0.4	0.2	0.2	0.1	0.1	0.1	0.1	0.1	0	0	0.4	0.2	0.3	0.2	0.3
6	0.5	0.2	0.4	0	0.4	0.4	1	0.2	0.2	0.1	0.1	0.1	0.1	0.1	0	0	0.5	0.2	0.3	0.2	0.3
7	0.2	0.5	0.2	0	0.2	0.2	0.2	1	0.5	0.3	0.3	0.3	0.3	0.3	0	0	0.2	0.5	0.1	0.4	0.1
8	0.2	0.5	0.2	0	0.2	0.2	0.2	0.5	1	0.3	0.3	0.3	0.3	0.3	0	0	0.2	0.5	0.1	0.4	0.1
9	0.1	0.3	0.1	0	0.1	0.1	0.1	0.3	0.3	1	0.5	0.5	0.5	0.4	0	0	0.1	0.3	0.1	0.2	0.1
10	0.1	0.3	0.1	0	0.1	0.1	0.1	0.3	0.3	0.5	1	0.5	0.5	0.4	0	0	0.1	0.3	0.1	0.2	0.1
11	0.1	0.3	0.1	0	0.1	0.1	0.1	0.3	0.3	0.5	0.5	1	0.5	0.4	0	0	0.1	0.3	0.1	0.2	0.1
12	0.1	0.3	0.1	0	0.1	0.1	0.1	0.3	0.3	0.5	0.5	0.5	1	0.4	0	0	0.1	0.3	0.1	0.2	0.1
13	0.1	0.3	0.1	0	0.1	0.1	0.1	0.3	0.3	0.4	0.4	0.4	0.4	1	0	0	0.1	0.3	0.1	0.2	0.1
14	0	0	0	0	0	0	0	0	0	0	0	0	0	0	1	0	0	0	0	0	0
15	0	0	0	0	0	0	0	0	0	0	0	0	0	0	0	1	0	0	0	0	0
16	0.5	0.2	0.4	0	0.4	0.4	0.5	0.2	0.2	0.1	0.1	0.1	0.1	0.1	0	0	1	0.2	0.3	0.2	0.3
17	0.2	0.5	0.2	0	0.2	0.2	0.2	0.5	0.5	0.3	0.3	0.3	0.3	0.3	0	0	0.2	1	0.1	0.4	0.1
18	0.3	0.1	0.3	0	0.3	0.3	0.3	0.1	0.1	0.1	0.1	0.1	0.1	0.1	0	0	0.3	0.1	1	0.1	0.2
19	0.2	0.4	0.2	0	0.2	0.2	0.2	0.4	0.4	0.2	0.2	0.2	0.2	0.2	0	0	0.2	0.4	0.1	1	0.1
20	0.3	0.1	0.3	0	0.3	0.3	0.3	0.1	0.1	0.1	0.1	0.1	0.1	0.1	0	0	0.3	0.1	0.2	0.1	1

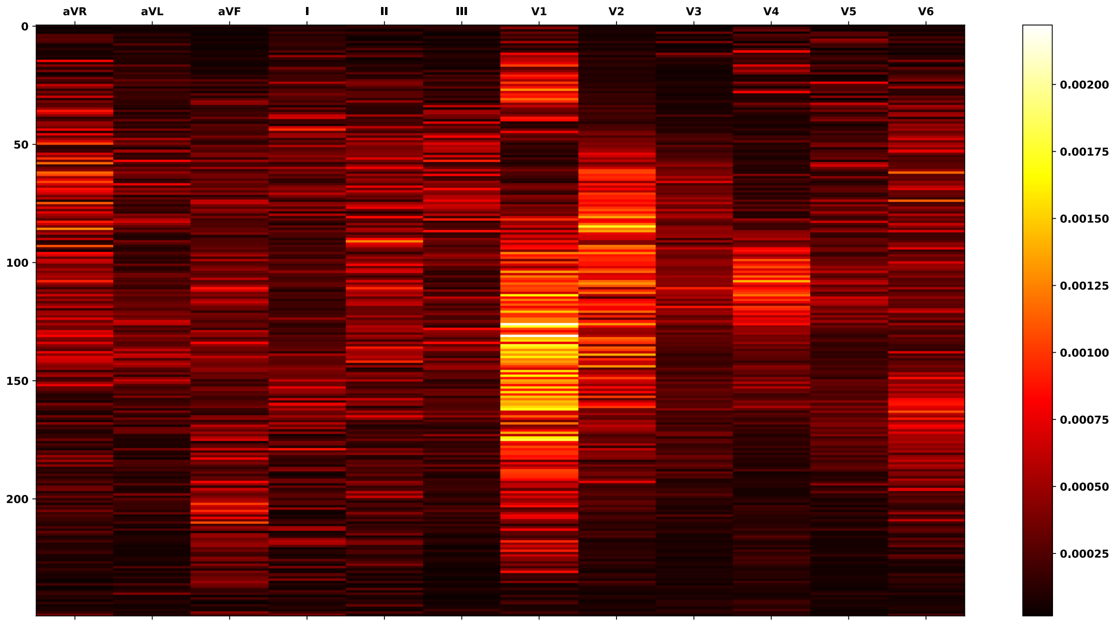

Figure 5: Feature importance heat map for classification scheme 1. X-axis denotes 12 leads and Y-axis denotes the sampling point. The most important features with bright colors in the map condense in the lead-V1, lead-V2, and lead-aVR sequentially. The top three important features are: in lead-V1 the voltage value at the 7th point after reference line; in lead-V1 the voltage value at the 2nd point after reference line; in lead-V1 the voltage value at the 51st point after reference line. The point at the reference line is counted as the first point. The reference line for each patient is the R-wave peak point at lead-II.

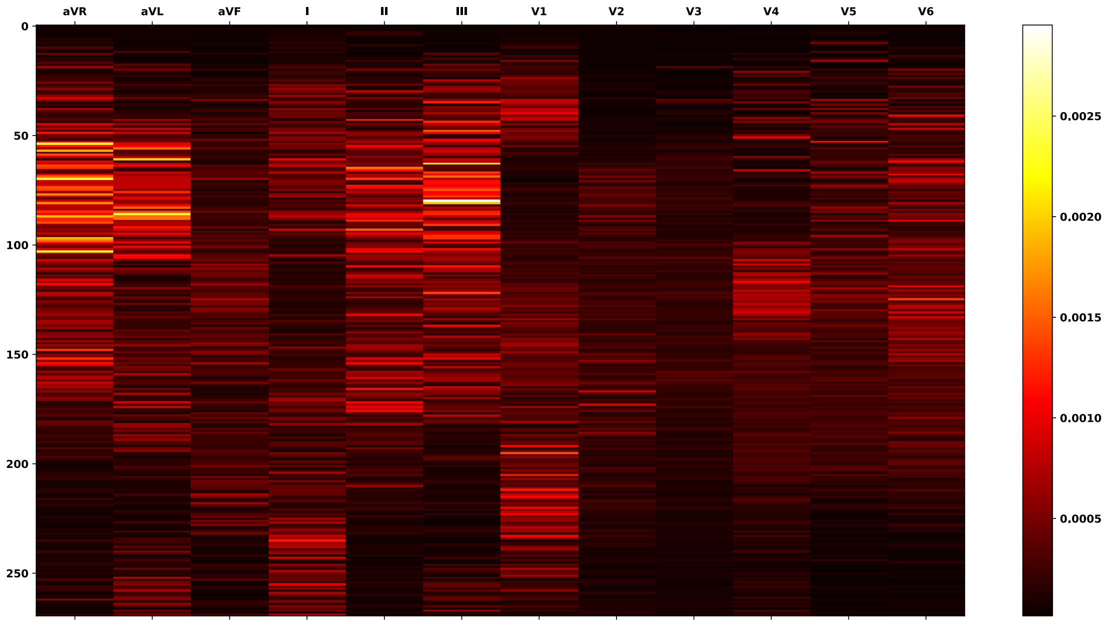

Figure 6: Feature importance heat map for classification scheme 2. X-axis denotes 12 leads and Y-axis denotes the sampling point. The most important features with bright colors in the map condense in the lead-III, lead-aVRL, and lead-aVR sequentially. The top three important features are: in lead-III the voltage value at the 55th point before the reference line; in lead-aVL the voltage value at the 49th point before reference line; in lead-aVR the voltage value at the 81st point before reference line. The point at the reference line is counted as the first point. The reference line for each patient is the R-wave peak point at lead-II.

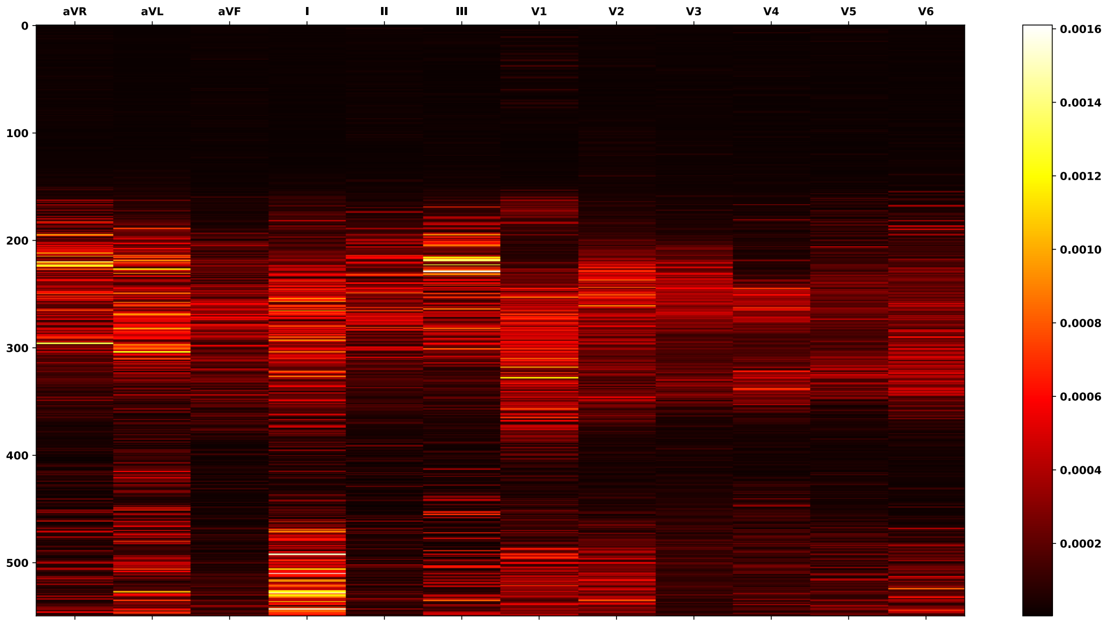

Figure 7: Feature importance heat map for classification scheme 3. X-axis denotes 12 leads and Y-axis denotes the sampling point. The most important features with bright colors in the map condense in the lead-I and lead-III sequentially. The top three important features are: in lead-I the voltage value at the 235th point after reference line; in lead I the voltage value at the 253th point after reference line; in lead-III the voltage value at the 46th point before the reference line. The point at the reference line is counted as the first point. The reference line for each patient is the R-wave peak point at lead-II.

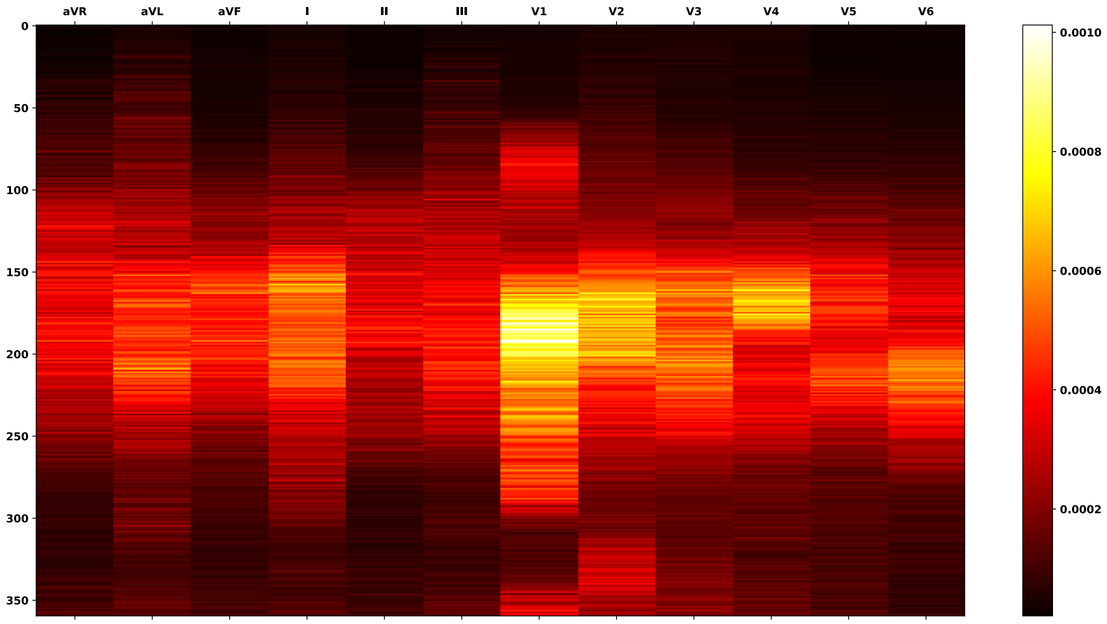

Figure 8: The feature importance heat map for classification scheme 4. X-axis denotes 12 leads and Y-axis denotes the sampling point. Heat map tells that lead-V1 plays the major role to predict 18 anatomical origins.The top three important features are: in lead-V1 the voltage value at the 11th point after reference line; in lead-V1 the voltage value at the 4th point after reference line; in lead V1 the voltage value at the reference.The point at the reference line is counted as the first point. The reference line for each patient is the R-wave peak point at lead-II.

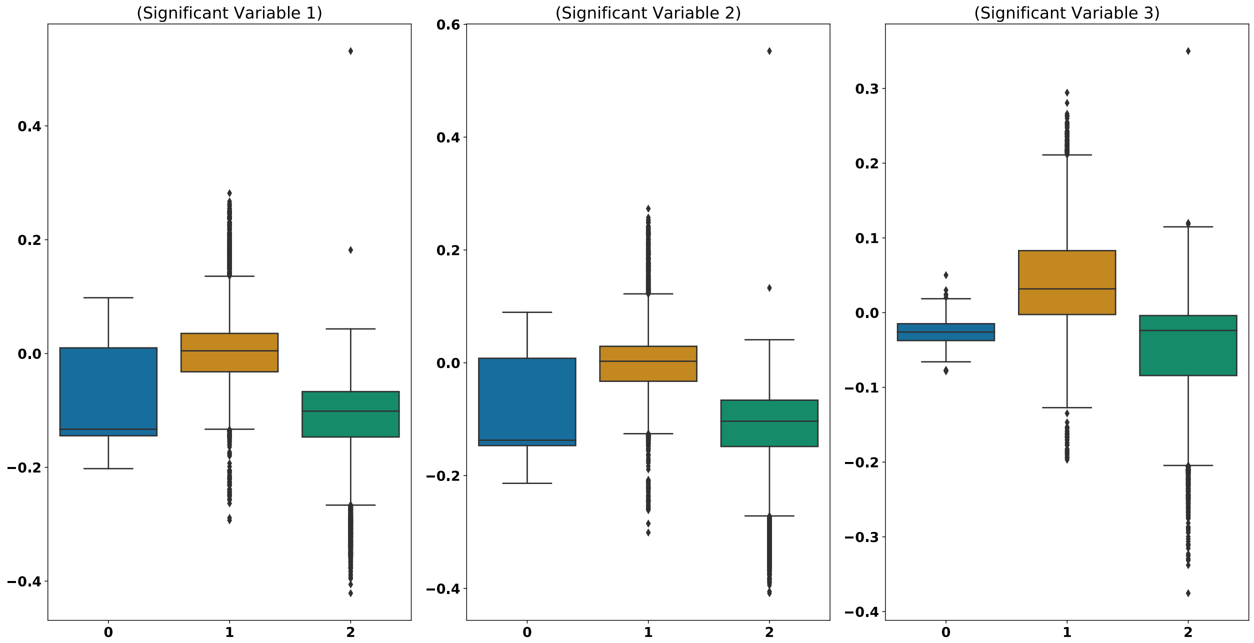

Figure 9: Univariate analysis of the first three important features for the classification scheme 1. Feature importance is used to measure the magnitude of variables impact in predicting the site of origin. The top three important variables were presented as below: Significant variable 1 = in lead-V1 the voltage value at the 7th point after reference line; Significant variable 2 = in lead-V1 the voltage value at the 2nd point after reference line; Significant variable 3 = in lead-V1 the voltage value at the 51st point after reference line. The point at the reference line is counted as the first point. The reference line for each patient is the R-wave peak point at lead-II. 0 = Epicardium of LV summit; 1 = LV endocardium; 2 = RV endocardium.

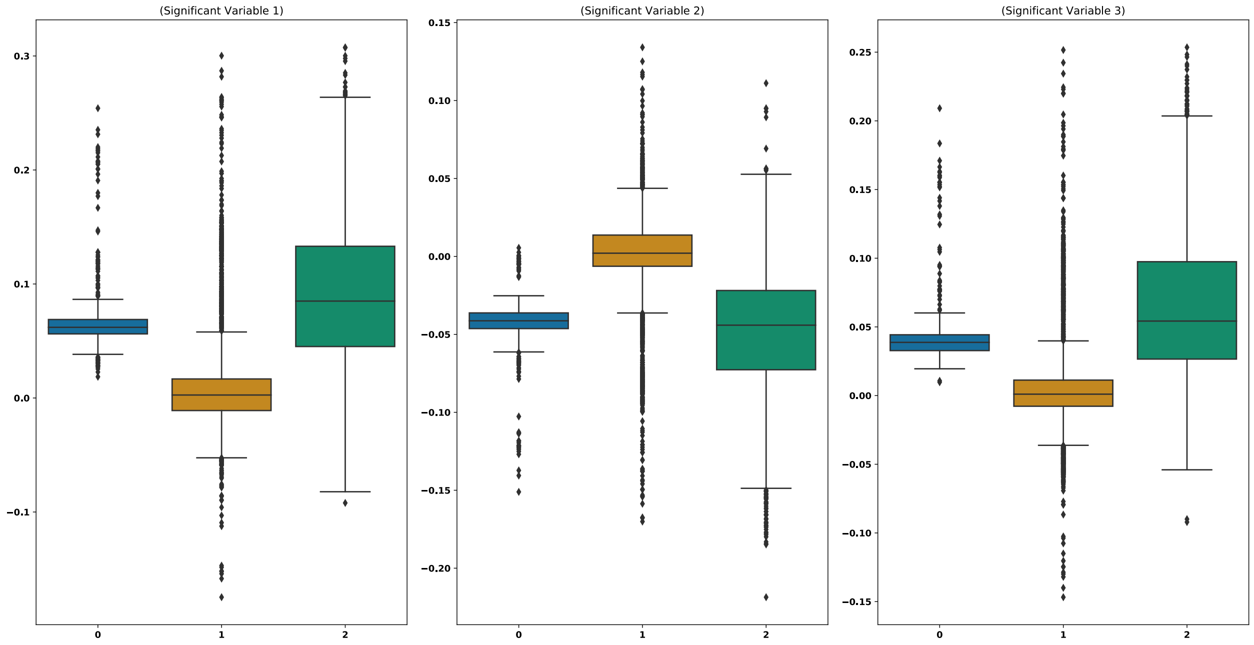

Figure 10: Univariate analysis of the first three important features for the classification scheme 2. Feature importance is used to measure the magnitude of variables impact in predicting the site of origin. The top three important variables were presented as below: Significant variable 1 = in lead-III the voltage value at the 55th point before the reference line; Significant variable 2 = in lead-aVL the voltage value at the 49th point before reference line; Significant variable 3 = in lead-aVR the voltage value at the 81st point before reference line. The point at the reference line is counted as the first point. The reference line for each patient is the R-wave peak point at lead-II. 0 = Epicardium of LV summit; 1 = Non-outflow tract endocardium; 2 = Outflow tract endocardium.

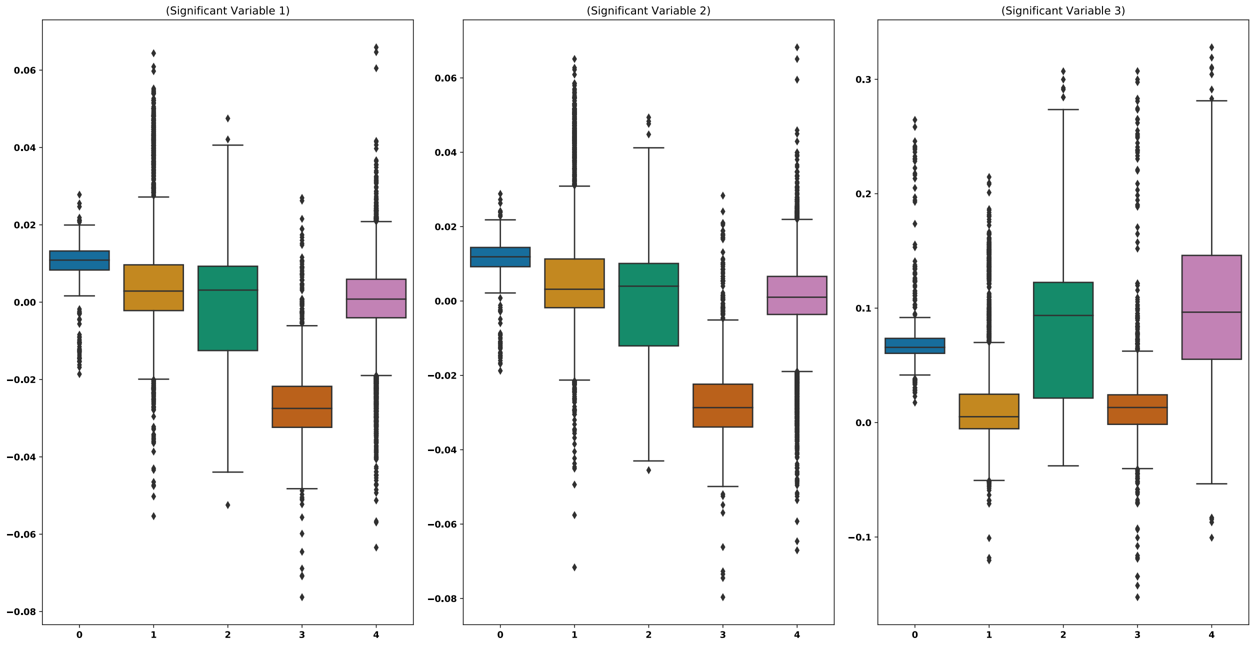

Figure 11: Univariate analysis of the first three important features for the classification scheme 3. Feature importance is used to measure the magnitude of variables impact in predicting the site of origin. The top three important variables were presented as below: Significant variable 1 = in lead-I the voltage value at the 235th point after reference line; Significant variable 2 = in lead I the voltage value at the 253th point after reference line; Significant variable 3 = in lead-III the voltage value at the 46th point before the reference line. The point at the reference line is counted as the first point. The reference line for each patient is the R-wave peak point at lead-II. 0 = Epicardium of LV summit; 1 = LV non-outflow tract endocardium; 2 = LVOT endocardium; 3 = RV non-outflow tract endocardium; 4 = RVOT endocardium. The abbreviations are as in Table 3.

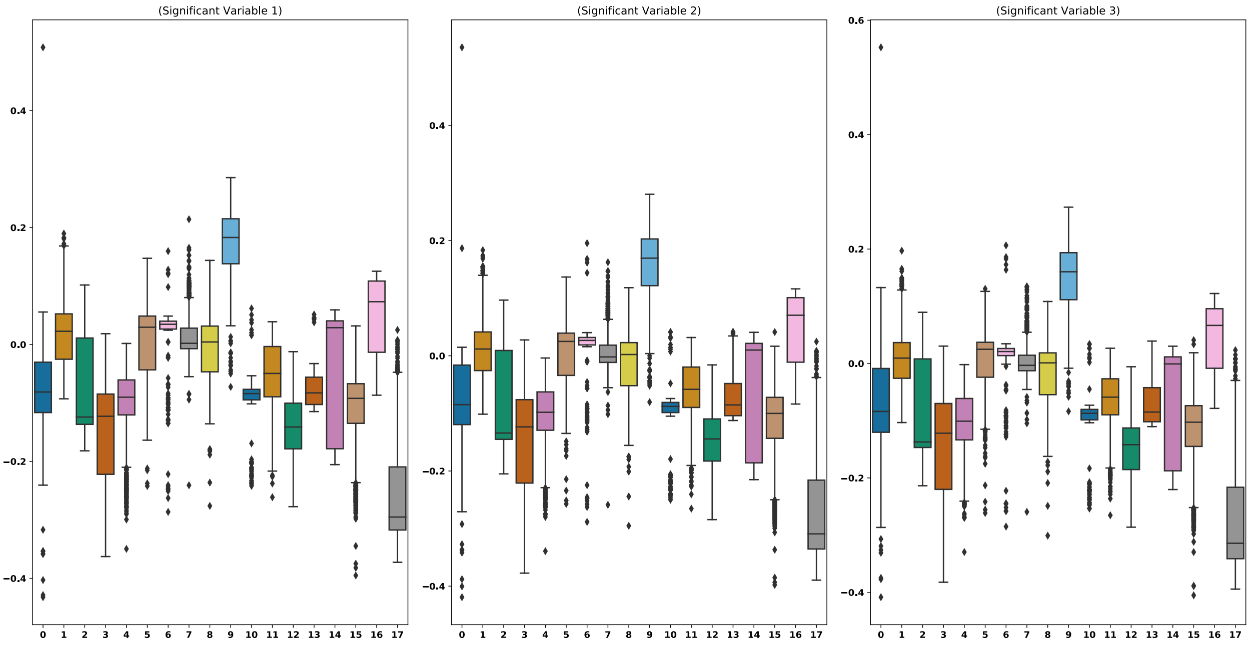

Figure 12: Univariate analysis of the first three important features for the classification scheme 4. Feature importance is used to measure the magnitude of variables impact in predicting the site of origin. The top three important variables were presented as below:Significant variable 1 = in lead-V1 the voltage value at the 11th point after reference line; Significant variable 2 = in lead-V1 the voltage value at the 4th point after reference line; Significant variable 3 = in lead V1 the voltage value at the reference line.The point at the reference line is counted as the first point. The reference line for each patient is the R-wave peak point at lead-II. 0 = AC; 1 = AMC; 2 = Epicardium of LV summit; 3 = RVOT free wall ; 4 = LC; 5 = LCC; 6 = LCC-RCC commissure; 7 = Left papillary muscle; 8 = Left septal; 9 = MV; 10 = Left His bundle; 11 = Right His bundle; 12 = RC; 13 = RCC; 14 = RAPM; 15 = Right septal; 16 = Summit; 17 = TV. The abbreviations are as in Table 4.

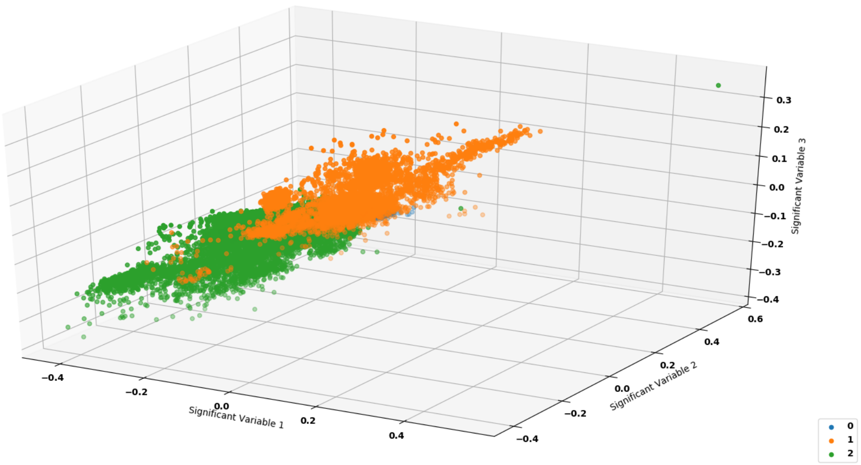

Figure 13: Multivariate analysis of the first three importance features for the classification scheme 1. 0 = Epicardium of LV summit; 1 = LV endocardium; 2 = RV endocardium. The definitions of significant variables are same with Figure 9. The reference line for each patient is the R-wave peak point at lead-II.

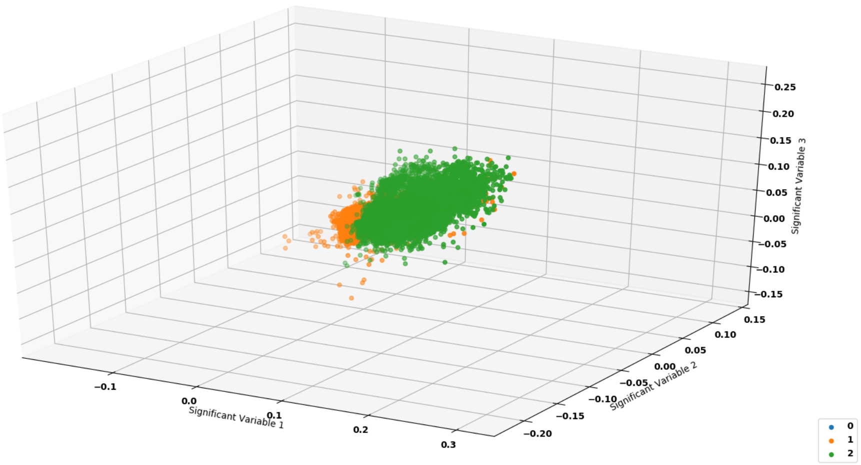

Figure 14: Multivariate analysis of the first three importance features for the classification scheme 2. 0 = Epicardium of LV summit; 1 = Non-outflow tract; 2 = Outflow tract. The definitions of significant variables are same with Figure 10.The reference line for each patient is the R-wave peak point at lead-II.

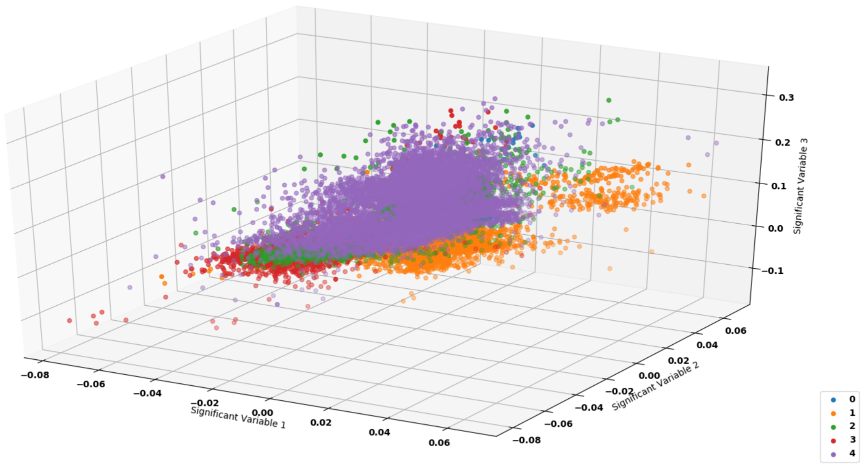

Figure 15: Multivariate analysis of the first three importance features for the classification scheme 3. 0 = Epicardium of LV summit; 1 = Left ventricular non-outflow tract; 2 = Left ventricular outflow tract; 3 = Right ventricular non-outflow tract; 4 = Right ventricular outflow tract. The definitions of significant variables are same with Figure 11.The reference line for each patient is the R-wave peak point at lead-II.

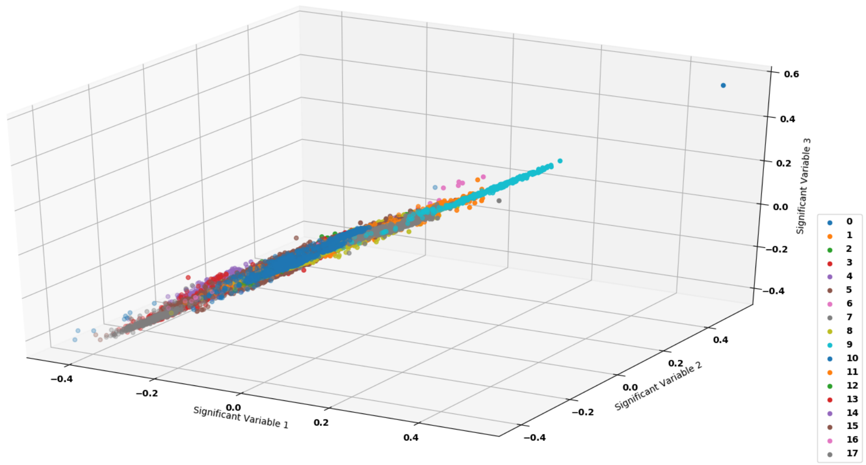

Figure 16: Multivariate analysis of the first three importance features for the classification scheme 4. 0 = AC; 1 = AMC; 2 = Epicardium of LV summit; 3 = RVOT free wall ; 4 = LC; 5 = LCC; 6 = LCC-RCC commissure; 7 = Left papillary muscle; 8 = Left septal; 9 = MV; 10 = Left His bundle; 11 = Right His bundle; 12 = RC; 13 = RCC; 14 = RAPM; 15 = RightSeptal; 16 = Summit; 17 = TV. The definitions of significant variables are same with Figure 12. The reference line for each patient is the R-wave peak point at lead-II. The abbreviations are as in Table 4.

Table 12: Comparison with prior studies to determine the origins of idiopathic ventricular arrhythmia. The first column represents the first author's name and the reference number in the main text. TZ = transition zone; SE = sensitivity; SP = specificity; PPV = positive predictive value; NPV = native predictive value; ACC = accuracy; AUC = area under curve.

Author	Patients	The study scope	ECG criteria/Algorithm	Reported performance in the article
This study	545	21 anatomical locations	machine learning algorithm and 0.16 second ECG data around R-wave peak point in lead-II	ACC 98.24 (97.36-98.71) F1-score 98.56 (97.88-99.12) adjusted ACC of 98.75 (98.35-99.38)
Xia (6)	275	RVOT and LVOT	The QRSi40 of identical precordial leads	ACC 90.7%, SE 84.4%, SP 93.3%
Yu (7)	75	Posterior RVOT, His, and RCC	the combination of R-wave amplitude in lead I and V2S/V3R index	Posterior RVOT: SE 96.2% SP 69.6% PPV 87.7% NPV 88.9% ACC 88% His: SE 100% SP 70% PPV 60% NPV 97.2% ACC 97.3% RCC: SE 52.9% SP 93.1% PPV 69.2% NPV 87.1% ACC 84%
Di (8)	184	RVOT and LVOT	V1-V3 transition index to predict RVOT	SE 93% SP 86% AUC 0.931 ACC 95%
Cheng (11)	94	RVOT and LVOT	a) R/S transition at lead V1/V2 predicts LVOT origin	SE 52.4% SP 92.1% PPV 72.6% NPV 85.3% ACC 84.2%
			b) R/S transition at lead V3 predicts RVOT origin	SE 39% SP 35.2% PPV 74.2% NPV 29.4% ACC 46.3%
			c) R/S transition at lead V4 or later predicts RVOT origin	SE 59.3% SP 93.1% PPV 94.6% NPV 46.7% ACC 68.3%
He (12)	488	RVOT and LVOT	Y=-1.15*(TZ)-0.494*(V2S/V3R)	SE 90% SP 87% AUC 0.88%
Xie (13)	75	RVOT and LVOT with left bundle branch block right inferior axis	R-wave amplitude $\geq$ 0.1 mV to predict LVOT	SE 75% SP 98% PPV 92.3% NPV 93% AUC 0.85%
Efimova (14)	105	RVOT and LVOT	A QRS-RVA (right ventricular apex) interval $\geq$ 0.49 ms predicts an LVOT origin. The QRS-RVA interval was measured from the onset of the QRS complex to the distal RVA signal.	SE 98%, SP 94.6%, PPR 94.1%, NPR 98.1%, ACC 96.1%
Yoshida (15)	207	RVOT and LVOT	V2S/V3R index $\leq$ 1.5 predicts LVOT origin	SE 89% SP 94% PPV 84% NPV 96%

Nakano (16)	63	RVOT and LVOT	a) R>S concordance in synthesized right-sided chest leads (Syn-V3R, Syn-V4R, Syn-V5R) predicts an LVOT origin	SE 100% SP 100%
			b) R/S index (>0.3): A ratio of R-wave amplitude to S-wave amplitude in leads V1 or V2 predicts an LVOT origin	SE 90% SP 98%
Cheng (17)	94	RVOT and LVOT	R-wave deflection interval in lead V3>80 ms and R-wave amplitude index in lead V1	SE 100% SP 83% PPV 85.7% NPV 100% ACC 91.7%
Yoshida (18)	112	RVOT and aortic sinus cusp	TZ index = TZ score of OTVT minus TZ score of a sinus beat	To aortic sinus cusp SE 88% SP 82% AUC 0.9
Betensky (19)	61	RVOT and LVOT	a) V2 transition ratio (defined as percentage R wave during VT divided by percentage R wave in SR) $\geq$ 0.6 predicts LVOT origin	SE 95% SP 100% PPV 100% NPV 95% ACC 91%
			b) PVC precordial transition later than SR transition predicts RVOT origin	SE 19% SP 100%
Zhang (22)	65	RVOT and LVOT	a) Transitional zone $\geq$ V4 predicts RVOT origin	SE 94.87% PPV 100%
			b) R-wave duration index <0.5 and R/S wave amplitude index <0.3 in V1/V2 predicts RVOT origin	SE 94.87% PPV 100%
Ito (25)	168	RVOT and LVOT	An ECG algorithm	SE 88% SP 95% PPV 88% NPV 96%
Kamakura (27)	35	RVOT and LVOT	(1) the QRS duration (>140 ms, A; $\leq$140 ms, P) and the R-wave pattern in leads II and III (RR' or Rr', A, R, P), (2) the QS wave amplitude in aVR and aVL (aVR$\geq$aVL, R; aVR<aVL, L), and (3) the r-wave amplitude in V1 and V2 (high, S; low, I)	Localized the origin with 80%, 86% (A-P), 80% (R-L), and 66% (S-I) accuracy.
Cardiologists (human experts)	545	21 anatomical locations	NA	SE 97.86% SP 81.72% F1-score 96.39% ACC 94.29%

Table 13: Hyperparameters table. DT = Decision Trees; KNN = K Nearest Neighbors; NC = Nearest Centroid; GNB = Gaussian Naive Bayesian; MNB = Multinomial Naive Bayesian; CNB = Complement Naive Bayesian; BNB = Bernoulli Naive Bayesian; LC = Linear Classifier; QDA = Quadratic Discriminant Analysis; MLR = Multinomial Logistic Regression; RRC = Ridge Regression Classifier; LCSGD = Linear Classifiers with Stochastic Gradient Descent; PAC = Passive Aggressive Classifier; SVC = Linear SVC; RF = Random Forest; ERT = Extreme Random Trees; GBT = Gradient Boosting Trees; EGBT = Extreme Gradient Boosting Trees.

Model Name	Hyperparameter Name	Hyperparameter Options
DT	criterion	'gini','entropy'
	splitter	'best','random'
	max_features	'auto','sqrt','log2',None
KNN	n_neighbors	15 31
	weights	'uniform','distance'
	algorithm	'ball_tree','kd_tree'
NC	shrink_threshold	0.01, 0.1, 0.2, 0.3
GNB	var_smoothing	$10^{-7\sim-12}$
MNB	alpha	0, 0.1, 0.5, 0.8,1
CNB	alpha	0, 0.1, 0.5, 0.8,1
BNB	alpha	0, 0.1, 0.5, 0.8,1
MLR	solver	'newton-cg','lbfgs','saga','sag'
RRC	alpha	1e-3, 1e-2, 1e-1, 1
	solver	'svd', 'cholesky', 'lsqr', 'sparse_cg', 'sag', 'saga'
LCSGD	loss	'hinge','log','modified_huber','squared_hinge','perceptron'
	alpha	1e-3, 1e-2, 1e-1, 1
	learning_rate	'constant','optimal','invscaling','adaptive'
	eta0	0.01, 0.001, 0.0001
PAC	C	0.001, 0.01, 0.1,1
	loss	'hinge','squared_hinge'
SVC	loss	'hinge','squared_hinge'
	C	0.001, 0.01, 0.1, 1
RF	n_estimators	300, 500, 800
	criterion	'gini', 'entropy'
	bootstrap	True, False
	max_features	'auto', 'sqrt', 'log2',None
ERT	n_estimators	300,500,800
	criterion	'gini', 'entropy'
	bootstrap	True, False
	max_features	'auto', 'sqrt', 'log2',None
GBT	loss	deviance, exponential
	learning_rate	0.1 , 0.01, 0.001, 0.1
	subsample	0.1, 0.5, 0.9
	n_estimators	300, 500, 800
	max_features	'auto', 'sqrt', 'log2', None
EGBT	tree_method	'auto', 'exact', 'approx', 'hist'
	grow_policy	'depthwise', 'lossguide'
	n_estimators	300, 500, 800
	learning_rate	0.001, 0.01
	max_depth	10, 15, 20, 50, 100
